# LncRNA PCAT1 activates AKT and NF-κB signaling in castration-resistant prostate cancer by regulating the PHLPP/FKBP51/IKKα complex

**DOI:** 10.1093/nar/gkz108

**Published:** 2019-02-18

**Authors:** Zhiqun Shang, Jianpeng Yu, Libin Sun, Jing Tian, Shimiao Zhu, Boya Zhang, Qian Dong, Ning Jiang, Amilcar Flores-Morales, Chawnshang Chang, Yuanjie Niu

**Affiliations:** 1 Tianjin Institute of Urology, the 2nd Hospital of Tianjin Medical University, Tianjin 300211, China; 2 Department of Urology, First Affiliated Hospital, Shanxi Medical University, Shanxi 030001, China; 3 Department of Health Science, Faculty of Health and Medical Sciences, University of Copenhagen, Copenhagen 2200, Denmark; 4 Department of Pathology and Urology, University of Rochester, Rochester, NY 14620, USA

## Abstract

In PTEN-deficient prostate cancers, AKT signaling may be activated upon suppression of androgen receptor signaling. Activation of AKT as well as NF-κB signaling involves a key regulatory protein complex containing PHLPP, FKBP51 and IKKα. Here, we report a critical role of lncRNA PCAT1 in regulating the PHLPP/FKBP51/IKKα complex and progression of castration-resistant prostate cancer (CRPC). Using database queries, bioinformatic analyses, as well as RIP and RNA pull-down assays, we discovered and validated that the lncRNA-PCAT1 perturbs the PHLPP/FKBP51/IKKα complex and activates AKT and NF-κB signaling. Expression of lncRNA-PCAT1 is positively linked to CRPC progression. PCAT1 binds directly to FKBP51, displacing PHLPP from the PHLPP/FKBP51/IKKα complex, leading to activation of AKT and NF-κB signaling. Targeting PCAT1 restores PHLPP binding to FKBP1 leading to suppression of AKT signaling. Preclinical study in a mouse model of CRPC suggests therapeutic potential by targeting lncRNA PCAT1 to suppress CRPC progression. Together, the newly identified PCAT1/FKBP51/IKKα complex provides mechanistic insight in the interplay between AKT, NF-κB and AR signaling in CRPC, and the preclinical studies suggest that a novel role for PCAT1 as a therapeutic target.

## INTRODUCTION

Prostate cancer (PCa) is the most commonly diagnosed malignancy among men and still ranks the second-leading cause of male cancer-related death in Western countries (1,2). With the development of magnetic resonance imaging (3,4) and prostate-specific antigen (PSA) screening (5,6), clinically significant PCa are being diagnosed at earlier stage. These patients are routinely treated with surgery and radiation with the intention to cure (7,8). Signaling mediated by the androgen receptor has an established role in the progression of PCa (9). Androgen-deprivation therapy (ADT) is the main systemic treatment for patients with locally advanced, biochemically recurrent PCa and metastatic prostate cancer. However, most patients initially sensitive to ADT will develop resistance to the treatment, and progression to castration-resistant prostate cancer (CRPC) is nearly inevitable. Metastatic CRPC is generally considered a lethal disease and currently managed by multiple lines of systemic therapies with moderate survival benefit.

The phosphatidylinositol 3-kinase (PI3K)/AKT pathway is one of the most prominent signaling pathways linked to PCa progression (10,11). PI3K activation results in phosphorylation of AKT and its downstream genes, including mammalian target of rapamycin (mTOR). Phosphorylated AKT (p-AKT), possessing a PH domain, can be considered as an indicator of PI3K/AKT pathway activation. The PI3K/AKT pathway may be antagonized by several phosphatases, such as phosphatase and tensin homolog gene (PTEN), and PH and leucine-rich repeat protein phosphatase (PHLPP) (12,13). Loss of PTEN is one of the most common genomic alterations in prostate cancer, and there is a reciprocal feedback between activation of AKT signaling and AR signaling (14). Activated AKT signaling regulates a variety of processes, especially cell proliferation and survival. In addition to AKT activation, it is also well known that nuclear factor κB (NF-κB )signaling is aberrantly activated in CRPC, with constitutively higher levels of NF-κB reported in castration-resistant cell lines when compared with androgen-dependent cell lines (15).

Non-coding RNAs (ncRNAs) are rising as key molecules, with the potential to serve as novel targets for CRPC and provide mechanistic insight in many uncharacterized aspects of CRPC. PCAT1, a long non-coding RNA (lncRNA), was first described in 2011 as a prostate-specific regulator of cell proliferation in prostate cancer (16). Zhang *et al.* found that PCAT1 could promote the progression of extrahepatic cholangiocarcinoma through activation of Wnt/β-catenin signaling (17). Other studies also demonstrated the oncogenic role of PCAT1 in gastric cancer, hepatocellular carcinoma, non-small cell lung cancer and bladder cancer (18–25). The prostate-specific role of PCAT1, particularly in relation to its role in CRPC, remains largely unknown.

In this study, we report a novel role of PCAT1 in CRPC. We reveal a critical role of PCAT1 in activating AKT and NF-κB signaling pathways. We revealed novel interaction between PCAT1 and a protein complex known to mediate AKT and NF-κB p65 activation, establishing PCAT1 as an emerging lncRNA functionally important in CRPC progression.

## MATERIALS AND METHODS

### Tissue specimens

Prostate tissue specimens used in this study were surgical specimens from patients with prostate cancer with complete clinicopathological data. ADPC specimens (*n* = 5) were acquired by radical prostatectomy, and CRPC specimens (*n* = 5) were acquired by transurethral resection of the prostate. These samples were paraffin-embedded and subjected to immunohistochemistry analysis and RISH assays with standard DAB staining protocols. In addition, eight ADPC samples acquired by radical prostatectomy and six CRPC samples acquired by transurethral resection of the prostate were fresh frozen in liquid nitrogen and processed for reverse transcription polymerase chain reaction (RT-PCR). Samples used in RT-PCR contained greater than 60% tumor content but were prepared without microdissection of tissues. All studies were approved by the Ethics Committee of the Second Hospital of Tianjin Medical University, and informed consent was obtained from all patients.

### Animal studies

The animal studies were approved by Tianjin Institute of Urology, Tianjin, China. Male nude mice (6 weeks old) were purchased from Beijing HFK Bioscience Co. Ltd. (Beijing, China). Subcutaneous tumor growth assays were performed with LNCaP-AI cells. After 2 weeks, the control set (*n* = 4) was injected with lentiviruses carrying control shRNA, and the trial set (*n* = 4) was injected with lentiviruses carrying lncRNA-PCAT1 shRNA in the inoculated site for 6 days. The growth of tumors was monitored everyday by measuring tumor size from the outside of mice skin. Volume was calculated with *V* = 1/2 × length × width^2^. After 6 days, the difference of tumor size in these two groups was visible, and the tumors were harvested under standard, institutionally approved processes. Tumor samples were paraffin fixed and processed for RISH analysis and immunohistochemistry analysis.

### Cell culture, cell lines and transfection

The parental androgen-dependent human prostate cancer cell line LNCaP and androgen independent cell line C4-2 were purchased from the American Type Culture Collection (ATCC, Manassas, VA, USA). The cell lines were maintained in RPMI-1640 medium (Gibco, Waltham, MA, USA) supplemented with 10% fetal bovine serum (Gibco), 100 ng/ml streptomycin and 100 U/ml penicillin (Gibco). For androgen deprivation, parental LNCaP cells were maintained in RPMI 1640 medium supplemented with 10% charcoal-stripped fetal bovine serum (BI, Cromwell, CT, USA). The LNCaP-AI line was generated following long-term culture of the parental LNCaP cells under androgen-deprived conditions. Cells were incubated at 37°C with 5% CO_2_. Transfection of LNCaP, LNCaP-AI and C4-2 cells reaching 50–70% confluency with siRNAs, and shRNA was performed using Lipofectamine 2000 (Invitrogen, Waltham, MA, USA) and X-tremeGENE HP Transfection Reagent (Roche, Indianapolis, IN, USA), respectively, according to the manufacturers’ instructions. Target sequences for siRNAs and shRNA used in this study included:

PCAT1 siRNA1 target sequence: ATACATAAGACCATGGAAAT, siRNA2 target sequence: GAACCTAACTGGACTTTAATT, PCAT1 shRNA target sequence: ATACATAAGACCATGGAAAT, FKBP51 shRNA target sequence: ACCTAATGCTGAGCT. PCAT1 overexpressed lentivirus was purchased from GENECHEM (Shanghai, China). PCAT1-MUT (Δ1001–1400 bp truncated mutant) overexpression lentivirus was purchased from GenePharma (Suzhou, China). GST-tagged FKBP51-WT (full-length) and GST-tagged FKBP51-MUT (Δ251–390AA truncated mutant) overexpression lentivirus were purchased from GenePharma.

### Analysis of public datasets

The Cancer Genome Atlas (TCGA) Prostate Adenocarcinoma datasets were retrieved from the cBioPortal (http://www.cbioportal.org/) for Cancer Genomics (26,27). DNA copy-number calls on 498 cases were determined using GISTIC 2.0: -2 = homozygous deletion; -1 = hemizygous deletion; 0 = neutral/no change; 1 = gain; 2 = high level amplification. Recurrence-free survival data calls on 492 cases and overall survival data calls on 498 cases were downloaded from the cBioPortal for Cancer Genomics survival module. The survival curves were constructed according to the Kaplan–Meier method and compared using the log-rank test. RNA-seq data for PCAT1 were retrieved from 498 ADPC cases and 118 mCRPC cases reported by the Cancer Genome Atlas (TCGA) (28) and the SU2C/PCF Dream Team (29). Relative expression differences for PCAT1 across the two cohorts were evaluated by FPKM values (fragments per kilobase of transcript per million mapped reads).

### Expression microarray analysis

LNCaP-AI and LNCaP cell lines were used for this microarray analysis. RNA quantity and quality were evaluated by NanoDrop ND-1000 (Thermo Scientific, Waltham, MA, USA), and RNA integrity was further assessed by standard denaturing agarose gel electrophoresis. Arraystar Human LncRNA Microarray V3.0 was designed for the global profiling of human LncRNAs and protein-coding transcripts, which was updated from the previous Microarray V2.0. A total of 30 586 LncRNAs and 26 109 coding transcripts were queried by this third-generation LncRNA microarray. Sample labeling and array hybridization were performed according to the Agilent One-Color Microarray-Based Gene Expression Analysis protocol (Agilent Technologies, Santa Clara, CA, USA) with minor modifications. Briefly, mRNA was purified from total RNA after removal of rRNA (mRNA-ONLY™ Eukaryotic mRNA Isolation Kit, Epicentre, Madison, WI, USA). Then, each sample was amplified and transcribed into fluorescent cRNA along the entire length of the transcripts without 3′ bias utilizing a random priming method (Arraystar Flash RNA Labeling Kit, Arraystar, Rockville, MD, USA). The labeled cRNAs were purified by RNeasy Mini Kit (Qiagen, Venlo, Netherlands). The concentration and specific activity of the labeled cRNAs (pmol Cy3/μg cRNA) were measured by NanoDrop ND-1000. Each labeled cRNA (1 μg) was fragmented by adding 5 μl of 10× Blocking Agent and 1 μl of 25× Fragmentation Buffer, heated at 60°C for 30 min, and finally mixed with 25 μl of 2× GE Hybridization buffer. The hybridization solution (50 μl) was dispensed into the gasket slide and assembled to the LncRNA expression microarray slide. The slides were incubated for 17 h at 65°C in an Agilent Hybridization Oven. Agilent Feature Extraction software (version 11.0.1.1) was used to analyze acquired array images. Quantile normalization and subsequent data processing were performed with using the GeneSpring GX v12.1 software package (Agilent Technologies). After quantile normalization of the raw data, differentially expressed LncRNAs with statistical significance between the two groups were identified through *P*-value/FDR and Fold Change filtering. Accession ID for the array data is GSE124291.

### RNA-Seq analysis

LNCaP-AI cells transfected with lncRNA PCAT1 shRNA and the scramble shRNA were used for the RNA-seq. RNA quantity and quality were evaluated by NanoDrop ND-1000, and RNA integrity further assessed by standard denaturing agarose gel electrophoresis. The sequencing libraries were generated using 1–2 μg of total RNA and the KAPA Stranded RNA-Seq Library Prep Kit from Illumina (San Diego, CA, USA) according to the manufacturer’s instructions. Briefly, the first step involved the enrichment of mRNA using NEBNext® Poly (A) mRNA Magnetic Isolation Module. Following purification, the RNA was fragmented into small pieces using divalent cations under elevated temperature. The cleaved RNA fragments were reverse-transcribed into first strand cDNA using reverse transcriptase and random primers, followed by second strand cDNA synthesis using DNA polymerase I and RNase H. These cDNA fragments were added a single ‘A’ base and followed by subsequent ligation of the adapter. The products were purified and enriched by polymerase chain reaction (PCR) to create the final cDNA library. The enrichment and size distribution of the libraries were tested by Agilent 2100 Bioanalyzer. The hybridization and cluster generation were performed on a cBot unit. The samples were single-end sequenced with a read length of 50 bp on an Illumina HiSeq 4000 sequencer. After pre-treatment of the raw reads (filtering and QC), sequencing reads were mapped against the human reference genome version 19 using the TopHat algorithm version 2.0.9 using Ensembl gene annotations version GRCh37.65. Further analysis was performed using the R statistical programming language version 2.15.0. After alignment to the genome, the expression level of genes was determined based on the value of FPKM (30), which was calculated by Ballgown (31). DEGseq package was applied to compare differentiated transcripts among sample based on FPKM values (fold-change ≥ 1.5, *P*-value ≤ 0.05). Genes positively regulated by AKT or NF-κB signaling were genes demonstrating downregulated expression after PCAT1 knockdown (PCAT1-knockdown versus PCAT1-shSCR) and based on KEGG pathways. Individual *P* values for each gene in the two signaling pathways were listed in Supplementary Table S4. Accession ID for the RNA-seq data is GSE124519.

### Quantitative RT-PCR analysis

The expression of lncRNA PCAT1 in eight ADPC frozen samples, six CRPC frozen samples, LNCaP-AI and C4-2 cells transfected with shRNA and siRNAs were detected with RT-PCR. Total RNA was isolated from cells using Trizol reagent (Invitrogen) and used for the first strand cDNA synthesis with the Reverse Transcription System (Roche) following the manufacturer’s protocol. Resulting cDNA was then analyzed by PCR using Applied Biosystems 7900 Real Time PCR System (Thermo Scientific) and SYBR Green PCR Master Mix (Roche) according to the manufacturers’ instructions. GAPDH and β-actin were used as an internal control. The relative expression of RNAs was calculated using the comparative *C*_t_ method. Primer sequences are listed below: PCAT1, forward 5′-TGAGAAGAGAAATCTATTGGAACC-3′ and reverse 5′-GGTTTGTCTCCGCTGCTTTA-3′; GAPDH, forward 5′-GGAGCGAGATCCCTCCAAAAT-3′ and reverse 5′-GGCTGTTGTCATACTTCTCATGG-3′; β-actin, forward 5′-GGGAAATCGTGCGTGACATTAAG-3′ and reverse 5′-TGTGTTGGCGTACAGGTCTTTG-3′. Separate primer sets used in mutant assays (Figure 4D and E) are listed below: PCAT1, forward 5′-CGCAAAGGAACCTAACTGGAC-3′ and reverse 5′-TTCATTGCACATCACAATCCG-3′; PCAT1-MUT, forward 5′-TTCCCATGTGCCTCTAAGTGC-3′ and reverse 5′-CCCGTTATGTTGACCAATGCC-3′.

### Antibodies

The following antibodies were used for immunoprecipitation, immunostainig and immunoblotting: FKBP51 (Abcam, Cambridge, UK, ab46002, 1:250 dilution for immunoblotting), AKT (Cell Signaling Technology, Danvers, MA, USA, #4691, 1:1000 dilution for immunoblotting, 1:300 dilution for immunohistochemistry), p-AKT Ser473 (Cell Signaling Technology, #4060, 1:2000 dilution for immunoblotting, 1:100 dilution for immunohistochemistry), IKKα (Cell Signaling Technology, #2682, 1:1000 dilution for immunoblotting), NF-κB p65 (Cell Signaling Technology, #8242, 1:1000 dilution for immunoblotting, 1:800 dilution for immunohistochemistry), p-NF-κB p65 (phospho S536) (Abcam, ab86299, 1:5000 dilution for immunoblotting, 1:1000 dilution for immunohistochemistry), PHLPP (Abcam, ab71972, 1:5000 dilution for immunoblotting), cleaved caspase3 (R&D, Minneapolis, MN, USA, MAB835, 1:1000 dilution for immunoblotting), BCL-2 (SANTA CRUZ, Dallas, TX, USA, sc-783, 1:200 dilution for immunoblotting), Ki67 (Abcam, ab16667, 1:250 dilution for immunoblotting, 1:100 dilution for immunohistochemistry), PCNA (Cell Signaling Technology, #2586, 1:2000 dilution for immunoblotting, 1:2400 dilution for immunofluorescence), p-Erk1/2 (Thr202/Tyr204) (Cell Signaling Technology, #4370, 1:400 dilution for immunohistochemistry, 1:2000 dilution for immunoblotting), p-4E-BP1 (Thr37/46) (Cell Signaling Technology, #2855, 1:100 dilution for immunohistochemistry, 1:1000 dilution for immunoblotting), c-Myc (Abcam, ab56, 1:500 dilution for immunohistochemistry, 1:1000 dilution for immunoblotting), GST (Abcam, ab19256, 1:5000 dilution for immunoblotting).

### Colony assays

About 10^3^ LNCaP-AI stable PCAT1-knockdown cells and 10^3^ LNCaP-AI stable shSCR cells were seeded in six-well plate, with three replicates. At week 3, colonies were stained with 0.5% crystal violet, imaged and counted.

### RNA pull-down assay

RNA pulldown was performed with Pierce™ Magnetic RNA-Protein Pull-Down Kit (Thermo Scientific) following the manufacturer’s suggestions. LncRNA-PCAT1 were *in vitro* transcribed from pReceiver-B02 Expression Clone, and biotin-labeled with the Pierce™ RNA 3′ End Desthiobiotinylation Kit (Thermo Scientific). Two hundred micrograms of whole-cell lysates from LNCaP-AI cells, FKBP51 and IKKα recombinant proteins were incubated with 50 pmol of purified biotinylated transcripts for 1 h at 4°C with rotation, respectively; complexes were isolated with streptavidin magnetic beads (Thermo Scientific) and subjected to standard immunoblot analysis.

### RNA immunoprecipitation (RIP)

RNA immunoprecipitation (RIP) was performed using 10^7^cells as described (32). Briefly, cells were harvested and lysed in RIP Lysis Buffer (10 mM 4-(2-hydroxyethyl)-1-piperazineethanesulfonic acid (HEPES), 100 mM KCl, 5 mM MgCl_2_, 0.5% NP-40, 1m M 1,4-Dithiothreitol (DTT), 5 mM PMSF, supplemented with protease inhibitors cocktail, RNase inhibitors and Panobinostat) for 5 min at 4°C and followed by centrifugation at 13 000 × *g* for 20 min. The cleared supernatant was collected and protein concentration determined by BCA Assay. Meanwhile, 50 μl of Protein A Sepharose Beads were coated by 4 μg of FKBP51 antibodies or control IgG at 4°C overnight, and 50 μl Protein A Sepharose Beads were coated by 5 μg of GST antibodies at 4°C overnight for GST-RIP assays. The coated Beads were washed with Lysis Buffer and incubated with 500 μl of the cell lysate, under moderate agitation overnight at 4°C. In the next day, RNA/beads complex with NT2 buffer was washed five times and re-suspended in NT2 buffer supplemented with RNase-free DNase and Proteinase K. The RNA was isolated using TRZol kit (Invitrogen) according to the manufacturer’s protocol, cDNA was synthesized and subjected to RT-PCR analysis.

### Immunoprecipitation (IP) and immunoblot analysis

Cells were incubated with lysis buffer (5 mM HEPES, pH 7.4, 150 mM NaCl, 1% Triton X-100, 10 mM glycerol, 1 mM ethylenediaminetetraacetic acid, 2 mM Na_3_VO_4_, 5 mM PMSF) for 30 min at 4°C and followed by centrifugation for 15 min at 13 000 rpm. Protein concentration of the lysates was determined by BCA Assay. For each experiment, 500 μg of protein was combined with 2–3 μg FKBP51 antibodies (Abcam, ab46002) and incubated under moderate agitation overnight at 4°C, followed by incubation for an additional 2 h after adding 50 μl of protein A Sepharose beads. Beads were collected with the magnet and washed five times with lysis buffer, and resuspended in 2× SDS sample buffer. Following boiling for 5 min, immunoprecipitates were subjected to SDS-PAGE and standard immunoblotting process.

### Immunohistochemistry (IHC)

FFPE sections of 5 μm thickness were prepared on charged glass slides. After deparaffinization and rehydration, slides were immersed in 10 mM citrate buffer (pH 7.5) and microwaved at 750 W for 30 min for antigen retrieval. Endogenous peroxidase activity was blocked by adding 3% hydrogen peroxide. The sections were incubated with diluted antibodies followed by polymerconjugated horseradish peroxidase in a humidified chamber. Standard DAB staining was performed for chromogenic detection of the IHC targets.

### Cell growth assay

Cell growth was monitored by the 3-(4,5-dimethylthiazol-2-yl)-2,5-diphe-nyltetrazolium bromide proliferation assay. Each experiment was performed in triplicate and repeated at least three times.

### Immunofluorescence staining (IF) and RISH assay

Cells were grown on cover glasses and fixed with 4% paraformaldehyde in phosphate-buffered saline (PBS) for 10 min. After permeabilizition with 0.2% Triton X-100 for 10 min and incubation with blocking buffer (PBS with 5% bovine serum albumin) for 20 min, the cells were incubated with PCNA antibody (Cell Signaling Technology, #2586) overnight at 4°C, and then with Alexa-Fluor-labeled donkey anti-goat antibody or donkey anti-rabbit antibody at room temperature for 2 h. Cell nucleus was stained with 4′,6-diamidino-2-phenylindole (DAPI). The stained cover glasses were mounted on standard slides and examined under Olympus FV1000D microscope.

The RISH probe targeting PCAT1 was designed and synthesized by Advanced Cell Diagnostics (Newark, CA, USA), and detection of PCAT1 expression was performed using the RNAscope 2.5 (HD)-BROWN Assay (Advanced Cell Diagnostics) in accordance with the manufacturer’s instructions. For animal studies, specific RISH signal was identified as punctate dots, and expression of PCAT1 was quantified according to Rohit Mehra *et al.* (33) and Yajia Zhang *et al.* (34): ≤1 dot per 10 cells = 0, 1 to 3 dots per cell = 1, 4 to 9 dots per cell (few or no dot clusters) = 2, 10 to 14 dots per cell (<10% in dot clusters) = 3 and greater than 15 dots per cell (more than 10% in dot clusters) = 4. For each tissue sample, a cumulative RISH product score was calculated as the sum of the individual products of the expression level (0–4) and percentage of cells (0–100) (i.e. [*A*% × 0] + [*B*% × 1] + [*C*% × 2] + [*D*% × 3] + [*E*% × 4]; total range = 0–400). For each tissue sample, the RISH product score was averaged across three random fields.

### Bioinformatics analysis

The binding propensity of FKBP51 protein and lncRNA PCAT1 pairs was estimated via algorithms supplied by catRAPID and prediction of lncRNA–protein interactions (35–37). The catRAPID omics module, catRAPID signature module and catRAPID fragments module were used to predict RNA-binding regions of FKBP51 proteins and the interaction between FKBP51 protein and lncRNA-PCAT1. Computational prediction of associations between FKBP51 protein and lncRNA-PCAT1 was also used to generate a predicted value (38).

### Statistical analysis

Overall survival and recurrence-free survival trends and curves were calculated by the Kaplan–Meier method, and differences were evaluated using the log-rank test. Summary data were expressed as mean ± S.D. The Student’s *t* and ANOVA tests were used to compare experimental groups. A *P* value of ≤0.05 (two-sided) was considered to indicate a statistically significant difference. All statistical analysis was performed with SPSS 22 statistical software (SPSS, IBM Corporation, Armonk, NY, USA).

## RESULTS

### LncRNA PCAT1 expression is correlated with prostate cancer progression and development of castration resistance

**Figure 1.**
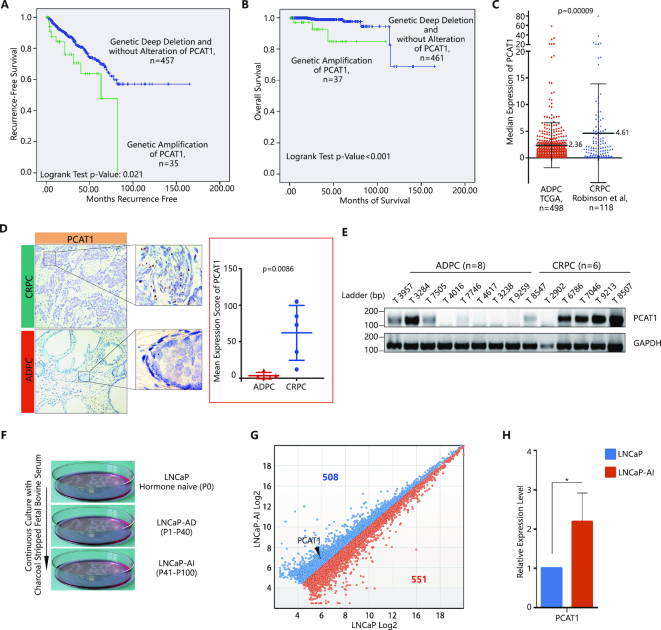
LncRNA-PCAT1 expression is correlated with castration-resistant prostate cancer. (**A**) Kaplan–Meier curve of the recurrence-free survival rates in prostate cancer patients with and without genetic amplification of PCAT1 (*P* = 0.021). Those without PCAT1 amplification included samples with deep deletion (*n* = 3) as well as those without alteration of PCAT1. The Cancer Genome Atlas data were retrieved from cBioPortal. (**B**) Kaplan–Meier curve of the overall survival rates in prostate cancer patients with and without genetic amplification of PCAT1 (*P* < 0.001). Those without PCAT amplification included samples with deep deletion (*n* = 3) as well as those without alteration of PCAT1. The Cancer Genome Atlas data were retrieved from cBioPortal. (**C**) Median expression of PCAT1 from two independent PCa patients’ RNA-seq data sets based on the value of FPKM. TCGA, The Cancer Genome Atlas; ADPC, androgen dependent prostate cancer; CRPC, castration-resistant prostate cancer. In dot plots, the center line is the median, with each dot depicting the FPKM value of each patient. The *P* value was determined by two-tailed *t-*tests. (**D**) RISH detection of PCAT1 expression in ADPC versus CRPC. Left panel: representative images; right panel: statistical analysis of five ADPC patient specimens and five CRPC patient specimens. (**E**) RT-PCR analysis of PCAT1 in fresh surgical specimens from patients with CRPC (*n* = 6) and ADPC (*n* = 8). GAPDH was used as a loading control. (**F**) Flowchart showing establishment of the androgen-independent LNCaP-AI cell line. The androgen-independent LNCaP-AI cell line was generated by long-term culture of androgen-dependent LNCaP cells in RPMI-1640 medium containing charcoal-stripped serum. LNCaP-AD, Androgen-dependent LNCaP cell line; LNCaP-AI, Androgen-independent LNCaP cell line. P, passage. (**G**) Scatter plots of lncRNAs significantly upregulated (blue) or downregulated (orange) in LNCaP-AI compared to LNCaP cells. *X* and *Y* axes are normalized signal values (log_2_ scaled) for each gene, with PCAT1 labeled by a red plot. (**H**) qRT-PCR detection of PCAT1 expression in LNCaP and LNCaP-AI cell lines, normalized by the level of GAPDH. A P-value of <0.05 was considered significant. *represents P < 0.05, **represents P < 0.01 and ***represents P < 0.001.

A number of recent studies implicated a role of lncRNA PCAT1 in post-transcriptional regulation of key cancer genes (16,25,39,40). However, the clinical relevance of PCAT1 expression in prostate cancer progression and castration-resistance remains largely unexplored. We retrieved public TCGA datasets from cBioPortal (26,27) and evaluated PCAT1 gene alterations in relation to recurrence-free survival data on 492 PCa patients (Supplementary Table S1) as well as overall survival data on 498 PCa patients (Supplementary Table S2). We found that PCa patients with PCAT1 gene amplification had worse recurrence-free and overall survival, respectively (Figure 1A and B), when compared to patients without PCAT1 amplification (inclusive of patients with normal copies or deletion of the PCAT1 locus). We further evaluated the RNA-seq data (FPKM value) from the TCGA androgen-sensitive PCa (ADPC) patients (*n* = 498) and CRPC patients from the SU2C/PCF Dream Team Study (*n* = 118) (Supplementary Table S3) (41). Although these clinical specimens demonstrated large variations in PCAT1 expression, PCAT1 expression was significantly higher in CRPC patients when compared with ADPC patients (Figure 1C). To further validate the data from the public domain, we performed RNA *in situ* hybridization (RISH) on CRPC (*n* = 5) and ADPC specimens (*n* = 5) collected in our institution. RISH results confirmed significantly higher PCAT1 levels in CRPC specimens (Figure 1D). The RISH results were consistent with RT-PCR findings that also confirmed higher expression of PCAT1 in the CRPC tissues (*n* = 6) when compared with ADPC tissues (*n* = 8) (Figure 1E).

To further evaluate the role of PCAT1 in castration-resistant growth of PCa, we generated and characterized an androgen-independent LNCaP-AI cell line by long-term culture of androgen-dependent LNCaP cells in RPMI-1640 medium containing charcoal-stripped serum (Figure 1F and Supplementary Figure S1A–C). The approach used to generate the line (Figure 1F) mimics the castration resistant condition for treating PCa (42,43), supporting the relevance of the LNCAP-AI cell line to CRPC. We conducted differential expression analysis of lncRNAs between LNCaP-AI cells and their parental LNCaP cells, and found that PCAT1 was significantly upregulated in LNCaP-AI cells (>2.0-fold) (Figure 1G). Results from qRT-PCR confirmed higher expression of lncRNA-PCAT1 in LNCaP-AI cells when compared to LNCaP cells (Figure 1H). Taken together, results from Figure 1A–H suggest that expression of lncRNA-PCAT1 is positively associated with CRPC progression.

### lncRNA PCAT1 activates AKT and NF-κB signaling in CRPC

**Figure 2.**
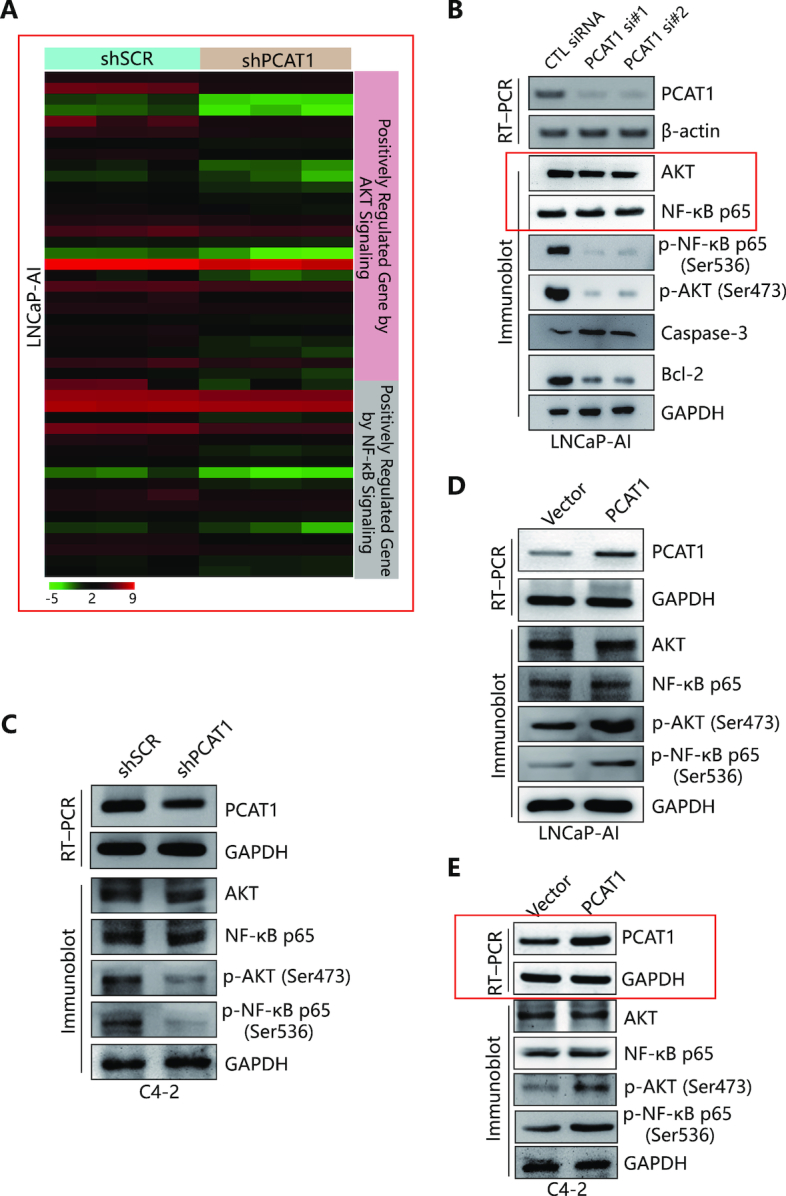
PCAT1 regulates AKT and NF-κB signaling pathways. (**A**) Heat map of key genes regulated positively by AKT or NF-κB signal pathways. These genes have decreased expression in PCAT1 depleted LNCaP-AI cells (*P* < 0.05). RNA-Seq data are displayed with log2 scaled FPKM (fragments per kilobase of transcript per million mapped reads) values for each gene in each sample. The gene names and other details for each specific gene were shown in Supplementary Table S4. (**B**) RT-PCR detection of PCAT1 and IB detection of indicated proteins in LNCaP-AI cells transfected with PCAT1 siRNAs. GAPDH was used as a loading control. (**C**) RT-PCR detection of PCAT1 and IB detection of AKT and NF-κB signaling molecules in C4-2 cells transfected with PCAT1 shRNAs. GAPDH was used as a loading control. (**D**) RT-PCR detection of PCAT1 in LNCaP-AI cell line with PCAT1 overexpression and IB detection of AKT and NF-κB signaling molecules. GAPDH was used as a loading control. (**E**) RT-PCR detection of PCAT1 in C4-2 cell line with PCAT1 overexpression and western blotting analysis of indicated proteins expression in PCAT1-overexpressed C4-2 cells. GAPDH was used as a loading control.

Given the putative role of lncRNA PCAT1 in CRPC, we examined the mRNA expression profiles in LNCaP-AI cells after knockdown of lncRNA-PCAT1 with shRNA. Strikingly, RNA-seq analysis revealed suppression of phosphoinositide 3-kinase (PI3K)/AKT and NF-κB signal pathways downstream targets as a result of PCAT1 knockdown (Figure 2A and Supplementary Table S4). AKT and NF-κB signal pathways are known to be constitutively activated in androgen-independent prostate cancer cell lines (44,45). In our cell line models, we have confirmed increased AKT and NF-κB p65 activities in the LNCaP-AI cell line when compared with the parental LNCaP line (Supplementary Figure S1D). To confirm the link between PCAT1 expression and activation of the AKT and NF-κB pathways, we further tested the expression of phosphorylated AKT (p-AKT), phosphorylated NF-κB p65 (p-NF-κB), caspase-3 (46,47) and B-cell lymphoma 2 (Bcl-2) (48–50) after siRNA knockdown of PCAT1 in LNCaP-AI cells. Depletion of PCAT1 resulted in a significant decrease of phosphorylated AKT, phosphorylated NF-κB p65 and Bcl-2 proteins, as well as increased caspase-3 protein levels (Figure 2B) without affecting the total protein levels, further confirming the inhibition of AKT and NF-κB signal pathways by PCAT1 knockdown. Similar results were observed in the C4-2 cell line, in which stable knockdown of PCAT1 resulted in a decrease of phosphorylated AKT and NF-κB p65 without altering the total AKT and NF-κB p65 protein levels (Figure 2C). We then evaluated the effect of PCAT1 overexpression in these cell lines. PCAT1 overexpression in LNCaP-AI and C4-2 cell lines further increased the levels of p-AKT and p-NF-κB p65 without affecting total AKT and NF-κB p65 levels (Figure 2D and E). These findings consistently support a role of PCAT1 in the activation of AKT and NF-κB signal pathways in CRPC by increasing phosphorylation level of AKT and NF-κB p65.

### lncRNA PCAT1 interacts with FKBP51 that mediates AKT and NF-κB signaling

A previous study revealed that dephosphorylation of p-AKT at S473 by phosphatase PHLPP requires FK506-binding protein 51 (FKBP51). In this process, FKBP51 protein acted as a scaffolding protein for the interaction between AKT and PHLPP to exert negative role for AKT signaling (51). FKBP51 is also known to interact with the nuclear factor IκB kinase α subunit (IKKα) to activate NF-κB signaling (52–55). Given the established interaction of FKBP51 with PHLPP and IKKα, we sought to dissect the mechanistic role of PCAT1 in AKT and NF-κB signaling by focusing on the interaction between lncRNA PCAT1 and FKBP51.

**Figure 3.**
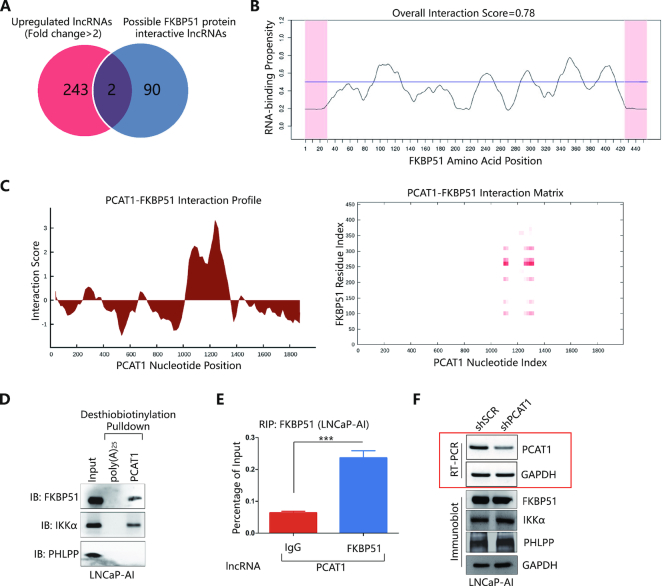
Bioinformatic identification and biochemical characterization of PCAT1-associated proteins. (**A**) Commonly upregulated (fold change > 2.0-fold, *P* < 0.01) lncRNAs (*n* = 245) in our Arraystar Human LncRNA Microarray V3.0 data (details in Supplementary Table S5) and possible lncRNAs (*n* = 92) interacted with FKBP51 protein predicted by the catRAPID omics module. (**B**) CatRAPID signature module prediction of the RNA-binding propensity for FKBP51 protein followed by prediction of RNA-binding regions. Overall interaction scores above 50% indicate propensity to bind. (**C**) CatRAPID fragments module prediction of the interaction profile and matrix between FKBP51 protein and PCAT1. (**D**) IB detection of proteins retrieved by *in vitro*-transcribed desthiobiotinylated PCAT1 from LNCaP-AI cell lysates. (**E**) RIP detection of the interaction between FKBP51 and PCAT1 by FKBP51 antibody in LNCaP-AI cells. The level of PCAT1 was determined by qRT-PCR and normalized by the input levels. A P-value of <0.05 was considered significant. *represents P < 0.05, **represents P < 0.01 and ***represents P < 0.001. (**F**) RT-PCR detection of PCAT1 in LNCaP-AI cell line after PCAT1 knockdown and IB detection of FKBP51, IKKα and PHLPP proteins expression after transfection with lentiviruses carrying PCAT1 shRNA in LNCaP-AI cells.

We first evaluated the possible interaction between PCAT1 and FKBP51 through bioinformatic approaches. Interestingly, among the 245 upregulated lncRNAs (Fold change > 2.0-fold, *P* < 0.01) in our lncRNA array data (Supplementary Table S5), only two lncRNAs, one of which was PCAT1, were predicted by the catRAPID omics module to interact with FKBP51 (Figure 3A and Supplementary Table S5). Results from catRAPID signature, an algorithm module in catRAPID server, revealed an overall FKBP51/PCAT1 interaction score of 0.78 (Figure 3B). Next, catRAPID fragments, another algorithm based on individual interaction propensities of polypeptide and nucleotide sequence fragments, further revealed that the 1093–1174 and 1249–1369 nucleotide positions of the PCAT1 sequence may bind to the 251–302 amino acid residues of the FKBP51 protein with high propensities (Figure 3C). The potential PCAT1/FKBP51 interaction is further supported by the matrix multiplication analysis showing a high score of 91.0624 for the interacting RNA–protein pairs (Supplementary Figure S1E) (38). Interestingly, the predicted interaction domain overlapped with the C-terminal tetratricopeptide repeat (TPR) domains of the FKBP51 protein (56) that was previously implicated in FKBP51/PHLPP interaction (51).

To confirm the PCAT1/FKBP51 interaction biochemically, we performed RNA pull-down assay using *in vitro*-transcribed biotinylated PCAT1 RNA and detected binding between PCAT1 and FKBP51 as well as IKKα proteins in LNCaP-AI cells, even under high stringency wash conditions (Figure 3D). However, PCAT1 did not bind to the PHLPP protein in LNCaP-AI cells (Figure 3D), suggesting that the PCAT1/FKBP51/IKKα complex may not include the PHLPP protein. RNA-binding protein immunoprecipitation (RIP) assay further confirmed the PCAT1/FKBP51 interaction in LNCaP-AI cells (Figure 3E). Knockdown of PCAT1 did not affect FKBP51, IKKα and PHLPP protein levels (Figure 3F).

### LncRNA–PCAT1–FKBP51 interaction reconfigures FKPB51–IKKα–PHLPP protein complex in CRPC

**Figure 4.**
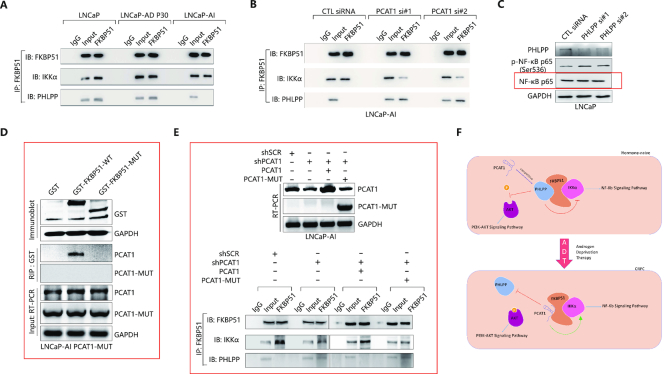
PCAT1/FKBP51 interaction perturbs FKPB51/IKKα/PHLPP protein complex in CRPC. (**A**) IP and IB detection of FKBP51/IKKα and FKBP51/PHLPP interactions in LNCaP cells, LNCaP-AD P30 cells and LNCaP-AI cells. LNCaP-AD, Androgen-dependent LNCaP cell line; LNCaP-AI, Androgen-independent LNCaP cell line. (**B**) IP and IB detection of FKBP51/IKKα and FKBP51/PHLPP interactions in LNCaP-AI cells transfected with indicated siRNAs. (**C**) Western blotting analysis of indicated proteins in LNCaP cells with PHLPP knockdown via PHLPP siRNAs. (**D**) Lack of PCAT1/FKBP51 interaction involving the truncation mutants. GST-tag (GST), GST-tagged full-length FKBP51 (GST-FKBP51-WT) and GST-tagged FKBP51 truncated mutant (GST-FKBP51-MUT) (Δ251–390AA) were transfected into PCAT1-MUT overexpressed LNCaP-AI cells (LNCaP-AI PCAT1-MUT). RIP assays were performed with the GST-tag antibody, and the levels of PCAT1 and PCAT1-MUT were determined by RT-PCR. PCAT1-MUT, PCAT1 truncated mutant (Δ1001–1400bp). (**E**) Overexpression of PCAT1, but not the mutant PCAT1, rescues the effect of PCAT1 silencing. PCAT1-truncated mutant (PCAT1-MUT) (Δ1001–1400bp) and wild-type PCAT1 were transfected into LNCaP-AI shPCAT1 stable cell line, and expression of PCAT1 and PCAT1-MUT were detected by RT-PCR (upper panel). FKBP51/IKKα and FKBP51/PHLPP interactions were determined in these cells by IP and IB assays. (**F**) A model for PCAT1-mediated activation of AKT and NF-κB signal pathways by perturbing FKPB51/IKKα/PHLPP protein complex in the progression from ADPC to CRPC after androgen deprivation therapy.

Given that the predicted PCAT1/FKBP51 interaction domain involves the C-terminal tetratricopeptide repeat (TPR) domains of the FKBP51 protein known to interact with PHLPP, PCAT1 may compete with PHLPP to interact with FKBP51. We evaluated FKPB51/IKKα/PHLPP protein interactions to determine whether these interactions differ in androgen-sensitive and androgen-independent cell lines. Immunoprecipitation (IP) and immunoblot (IB) results showed that while FKBP51/ IKKα interactions did not change in the ADPC and CRPC cell lines, the PHLPP protein binds to FKBP51 proteins specifically in LNCaP and LNCaP-AD (P30) cells, but not in LNCaP-AI cells that have higher PCAT1 expression (Figure 4A), suggesting PHLPP is displaced by PCAT1 in the absence of androgen. Knockdown of PCAT1 in LNCaP-AI cells restored FKBP51/PHLPP protein interaction (Figure 4B). Knockdown of PCAT1 also weakened FKBP51/IKKα interaction (Figure 4B), though lack of PCAT1 had minimal effect on the expression of FKBP51, PHLPP and IKKα (Figure 3F). Knockdown of PHLPP, however, resulted in elevated p-NF-κB p65 (Figure 4C).

### Further confirmation of PCAT1/FKBP51 interaction

To further confirm the specific sites of PCAT1/FKBP51 interaction predicted by bioinformatic analysis, PCAT1-truncated mutant (PCAT1-MUT) (Δ1001–1400bp) (Supplementary Figure S1F) was created and transfected into LNCaP-AI cells. In contrast to the wild-type PCAT1 (Figure 2D), PCAT1-MUT had minimal effect on AKT signaling and its downstream targets, including phosphorylated 4E-BP1 (p-4E-BP1 (Thr37/46)) and phosphorylated Erk1/2 (p-Erk1/2 (Thr202/Thr204)) (Supplementary Figure S1G). In addition, NF-κB signaling and the expression of its downstream gene, c-Myc, were not elevated in PCAT1-MUT overexpressed LNCaP-AI cells (Supplementary Figure S1G). These results suggested that mutant PCAT1 had no impact on AKT and NF-κB signaling, confirming the importance of the FKBP51 interaction mediated by the predicted PCAT-1 interaction sequences.

Next, GST-tag (GST), GST-tagged full-length FKBP51 (GST-FKBP51-WT) and GST-tagged FKBP51-truncated mutant (GST-FKBP51-MUT) (Δ251–390AA) (Supplementary Figure S1H) were created and transfected into LNCaP-AI cells with both endogenous PCAT-1 and transfected PCAT1-MUT (LNCaP-AI PCAT1-MUT), allowing assessment of interaction between the wild-type and mutant PCAT1/FKBP51 in the same cellular context (Figure 4D). RIP assays performed with GST-tag antibody confirmed interaction of wild-type FKBP51 with the wild-type PCAT1 only, and no interaction was detected between the mutant FKBP51 or PCAT1 with the respective wild-type partners, further confirming the essential role of the specific binding sites in PCAT1/FKBP51 interaction (Figure 4D).

To rescue the effect PCAT-1 silencing, PCAT1 and PCAT1-MUT were transfected into LNCaP-AI shPCAT1 cells respectively, and FKBP51/IKKα/PHLPP interactions were evaluated by IP and IB assays. Efficiency of PCAT1 and PCAT1-MUT overexpression was validated by RT-PCR results (Figure 4E). IP and IB results revealed that FKBP51/PHLPP interactions were abolished and FKBP51/ IKKα interactions were strengthened by PCAT1 overexpression, but these interactions were not affected by PCAT1-MUT overexpression (Figure 4E). Therefore, expression of wild-type PCAT-1, but not PCAT1-MUT, rescued the effect of PCAT-1 silencing (Figure 4E).

### Proposed model for PCAT1 in regulation of the FKPB51–IKKα–PHLPP protein complex

Therefore, our collective data support a model in which PCAT1/FKBP51 complex in CRPC accelerates the recruitment of IKKα with less obstruction compared with the PHLPP/FKBP51 complex in ADPC (see detailed mechanistic diagram in Figure 4F). With competitive inhibition of interaction between FKBP51 and PHLPP in CRPC, PCAT1 binds to FKBP51 directly to relieve the negative regulation of AKT signaling by PHLPP, and PCAT1/FKBP51 binding further stabilizes FKBP51/IKKα complex leading to increased NF-κB signaling (Figure 4F).

### LncRNA PCAT1 mediates cell growth in castration-resistant cell lines

**Figure 5.**
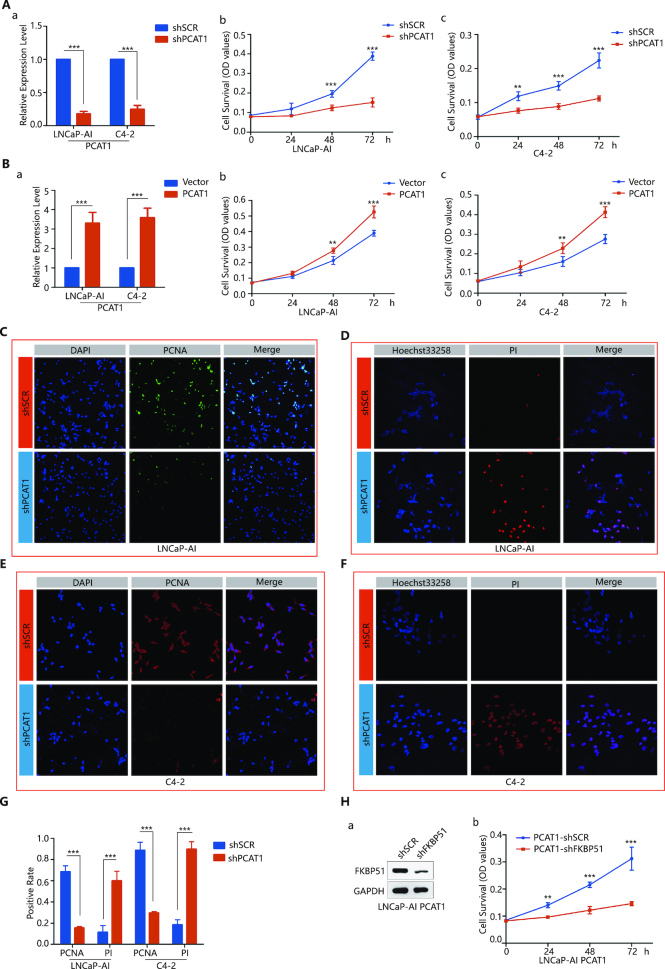
PCAT1 functions through the PCAT1/FKBP51/IKKα complex to promote CRPC progression. (**A**) MTT assays in LNCaP-AI and C4-2 cells infected with lentiviruses carrying shPCAT1. Left panel: qRT-PCR detection of PCAT1 expression after transfection with shPCAT1; right panel: cell growth assessed daily for 3 days using an MTT assay. Data were obtained from three independent experiments with samples in triplicate. One-way analysis of variance (ANOVA) and paired Student’s *t*-test was carried out using SPSS 22 statistical software. A *P*-value of <0.05 was considered significant. *represents *P* < 0.05, **represents *P* < 0.01 and ***represents *P* < 0.001. (**B**) MTT assays in LNCaP-AI and C4-2 cells infected with lentiviruses overexpressing PCAT1. Left panel: qRT-PCR detection of PCAT1 expression after overexpression of PCAT1; right panel: cell growth was assessed daily for 3 days using an MTT assay. A P-value of <0.05 was considered significant. *represents P < 0.05, **represents P < 0.01 and ***represents P < 0.001. (**C**) Immunofluorescence assays in PCAT1-deficient LNCaP-AI cells. For each group, representative images were randomly chosen under fluorescent microscopy with 200-fold magnification. (**D**) Hoechst33258-PI Staining assays in PCAT1-deficient LNCaP-AI cells. For each group, representative images were randomly chosen under fluorescent microscopy with 200-fold magnification. (**E**) Immunofluorescence assays in PCAT1-deficient C4-2 cells. For each group, representative images were randomly chosen under fluorescent microscopy with 200-fold magnification. (**F**) Hoechst33258-PI Staining assays in PCAT1-deficient C4-2 cells. For each group, representative images were randomly chosen under fluorescent microscopy with 200-fold magnification. (**G**) Statistical analysis of Figure 5C–F. A P-value of <0.05 was considered significant. *represents P < 0.05, **represents P < 0.01 and ***represents P < 0.001. (**H**) IB detection of FKBP51 and MTT assay detection of cells growth after FKBP51 knocked down in PCAT1 overexpressed LNCaP-AI cells. A P-value of <0.05 was considered significant. *represents P < 0.05, **represents P < 0.01 and ***represents P < 0.001.

To further investigate the biological function of PCAT1/FKBP51 in CRPC, we first used shRNA to knock down PCAT1 in the LNCaP-AI and C4-2 cell lines (Figure 5A). Knockdown of PCAT1 in these androgen-independent cell lines resulted significant inhibition of cell growth (Figure 5A). Consistent with the results in Figure 5A, colony assays also suggested the inhibition of cell growth in LNCaP-AI cells after PCAT1-knockdown (Supplementary Figure S1I). Conversely, significantly increased cell growth was detected in the same cell lines after PCAT1 overexpression (Figure 5B). The functional impact of PCAT1 in androgen-independent cell growth was further confirmed using another growth assay involving immunofluorescence (IF) analysis of PCNA. Confocal Laser Scanning Microscope (CLSM) results for the PCNA IF assay revealed that knockdown of PCAT1 in LNCaP-AI and C4-2 (Figure 5C, E and G) cells led to a marked decrease of PCNA positive rates, while the Hoechst33258-PI staining assay demonstrated a significant increase in the percentage of apoptotic cells after knockdown of PCAT1 in the LNCaP-AI and C4-2 (Figure 5D, F and G). While PCAT1 overexpression led to increased cell growth in LNCaP-AI cells (Figure 5B), knockdown of FKBP51 in these cells reversed the cell growth conferred by PCAT1 overexpression (Figure 5H), further supporting a critical role of PCAT1/FKBP51 interaction. And overexpression of PCAT1 was not altered by FKBP51-knockdown (Supplementary Figure S1J).

### Suppression of CRPC progression by targeting lncRNA PCAT1 in a preclinical mouse model

**Figure 6.**
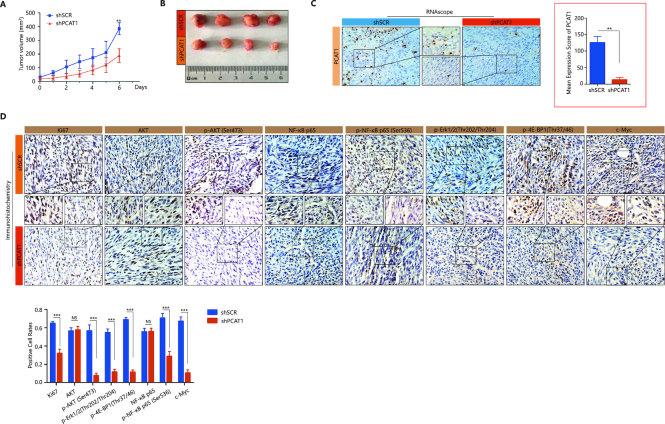
Preclinical studies targeting the lncRNA PCAT1 in a CRPC animal model. (**A** and **B**) Suppression of CRPC tumor growth in animals treated with PCAT1 shRNA (*n* = 4) versus scrambled shRNA (*n* = 4) for 6 days. Tumor sizes were measured for 6 days (A), at the time of tumor removal (B). A P-value of <0.05 was considered significant. *represents P < 0.05, **represents P < 0.01 and ***represents P < 0.001. (**C**) RISH detection of PCAT1 expression in the two indicated groups of mouse tumor specimens. For each group, six different fields were randomly chosen and counted under microscopy with 400-fold magnification. Representative images and statistical analysis are shown. Evaluation not blinded. A P-value of <0.05 was considered significant. *represents P < 0.05, **represents P < 0.01 and ***represents P < 0.001. (**D**) IHC staining of Ki67, p-AKT (Ser473), p-NF-κB p65 (Ser536) and indicated proteins in the two indicated groups of mouse tumor specimens. For each group, six different fields were randomly chosen and counted under microscopy with 400-fold magnification. Representative images and statistical analysis are shown. Positive rate (%) = number of positive cells/ number of total cells × 100%. Evaluation not blinded. A P-value of <0.05 was considered significant. *represents P < 0.05, **represents P < 0.01 and ***represents P < 0.001.

To explore the potential of targeting lncRNA PCAT1 in CRPC, we employed lentiviral injection into CRPC tumor xenografts. LNCaP-AI cells were xenografted into 6-week-old immunocompromised severe combined immunodeficiency (SCID) male mice (*n* = 8). The control set (*n* = 4) was injected with lentiviruses carrying control shRNA, while the treatment set (*n* = 4) was injected with lentiviruses carrying lncRNA-PCAT1 shRNA. Injections were carried out daily for 6 days. Knockdown of lncRNA PCAT1 during this short-term treatment period led to a significant decrease in the growth of the LNCaP-AI tumors (Figure 6A and B).

The efficiency of knockdown for PCAT1 was validated by RISH in the two sets of tumors (Figure 6C). In addition, the protein expression of Ki67, p-AKT, p-NF-κB p65 and AKT or NF-κB signaling downstream genes is significantly down-regulated in the treated animals when compared to the control animals (Figure 6D), suggesting on-target effect of PCAT1 knockdown consistent with *in vitro* cell-line findings.

## DISCUSSION

Previous studies implicated a role of lncRNA PCAT1 in multiple cancers. Prensner *et al.* built a computational framework for large-scale lncRNA analyses and described the active role of PCAT1 in promoting cell proliferation, with 370 downstream genes altered by PCAT1 involved in PCa progression (16). Shen *et al.* reported the potential significance of PCAT1 in auxiliary diagnosis of multiple myeloma due to elevated levels of serum lncRNA-PCAT1 (57). Bi *et al.* showed higher expression of PCAT1 in gastric cancers with poor prognosis, and inhibition of cell proliferation and invasion via regulating CDKN1A following PCAT1 knockdown (20). Wen *et al.* found that upregulation of PCAT1 increased cell proliferation and inhibited apoptosis in hepatocellular carcinoma (21). Zhao *et al.* determined the oncogenic role in non-small cell lung cancer progression (22). Clinical correlation between PCAT1 and cancer progression was also reported in a number of cancers (17,25,58,59). None of these previous studies, however, investigated the role of PCAT1 in a setting relevant to castration therapy that is the mainstay of prostate cancer treatment. To the best of our knowledge, the present study demonstrated for the first time a role of PCAT1 in promoting castration-resistant prostate cancer progression.

Our results are in agreement with previous studies consistently establishing PCAT1 as a lncRNA promoting cancer progression. Of importance, our novel findings revealed the molecular mechanisms underlying the role of PCAT1 in CRPC progression, particularly its role in regulating AKT and NF-κB signaling. Mulholland *et al.* (13) reported that prostate cancer driven by PTEN loss progressed through compensatory signaling pathways following androgen withdrawal or AR-targeted therapies. Carver *et al.* (14) reported reciprocal feedback regulation of PI3K and AR signaling in PTEN-deficient prostate cancers. Because PTEN is most commonly deleted tumor suppressor gene and AR is the most important therapeutic target in prostate cancer, these studies established the therapeutic importance of dissecting the relevant pathways. FKBP51 is androgen-responsive gene demonstrating decreased gene expression immediately following castration, leading to reduced PHLPP activity (which requires FKBP51 as a scaffolding protein), phosphorylation of AKT (S473) and activation of AKT signaling. The present study uncovered a critical role of PCAT1 in the interplay between AKT signaling and AR signaling. PCAT1 enhances AKT and NF-κB signaling to promote CRPC progression by displacing PHLPP, analogous to AKT activation resulting from AR inhibition reported in Carver *et al.* (14). In addition, we showed that PCAT1 competitively inhibited the binding of PHLPP to FKBP51, and facilitated the binding of IKKα-FKBP51 to enhance NF-κB signaling in CRPC.

One limitation of the study is the uncharacterized role of PCAT1 in CRPC cells with a functional PTEN due to our focus on cells with deficient PTEN. Prensner *et al.* reported that knockdown of PCAT1 resulted in a decrease of cell proliferation in normal LNCaP cells, and overexpression of PCAT1 in RWPE and Du145 cells resulted in increased cell proliferation (16,25,58). Interestingly, the PTEN-positive Du145 cells were not inhibited after knockdown of PCAT1 (16). The critical role of PCAT1 in regulating PHLPP/FKBP51/IKKα complex in CRPC with functional PTEN versus deficient PTEN warrants further investigation.

While our data support that PCAT1 expression is functionally essential for CRPC progression in our experimental models, another limitation of the study is lack of data supporting PCAT1 as an independent driver of androgen independence. PCAT1 overexpression may be a consequence of other changes in CRPC cells, and may be transcriptional regulated by other key drivers involved in CRPC progression. For example, Prensner *et al.* reported that the expression of PCAT1 was subject to regulation by PRC2 in VCaP cells (16). As such, whether PCAT1 expression alone is sufficient to drive CRPC progression remains uncharacterized. Nevertheless, our study findings demonstrated a novel role of PCAT1 with potential therapeutic implications for CRPC.

In summary, our study uncovered a critical role of lncRNA PCAT1 in CRPC. The PCAT1/FKBP51 and FKBP51/IKKα interactions that mediate AKT and NF-kB signaling may be regulated by PCAT1 expression. In this process, FKBP51 acts as a scaffolding protein regulating the function of PHLPP and IKKα that are perturbed by altered expression of PCAT1. This newly dissected process is relevant to CRPC because AR targeting changes the expression of FKBP51 and PCAT1. Study findings support future efforts in clinical development of PCAT1 as a therapeutic target and also a potential biomarker for castration-resistance prostate cancer.

## DATA AVAILABILITY

GEO accession ID for the array data is GSE124291. GEO accession ID for the RNA-seq data is GSE124519.

## Supplementary Material

Supplementary Data
