# Supplementary material for: LncRNA PCAT1 activates AKT and NF-κB signaling in castration-resistant prostate cancer by regulating the PHLPP/FKBP51/IKKα complex
Source: Nucleic Acids Res. 2019 Feb 18;47(8):4211–25. doi: 10.1093/nar/gkz108 (PMC6486551; doi:10.1093/nar/gkz108)
Supplement: Supplementary Data [file gkz108_supplemental_file.pdf]

Supplementary Material

Supplementary Figure Legends

Figure S1.

A. LNCaP cells are cultured in either normal medium or medium containing charcoal-stripped serum. Cell morphology was recorded. P, passage.
B. MTT assays of LNCaP, LNCaP-AD and LNCaP-AI cells. LNCaP-AD, Androgen-dependent LNCaP cell line; LNCaP-AI, Androgen-independent LNCaP cell line.
C. MTT assays of LNCaP-AI cells after treatment with Bicalutamide (10uM) or MDV3100 (10uM).
D. IB detection of indicated proteins level during the establishment of the LNCaP-AI cell line.
E. Computational prediction of interaction score between PCAT1 and FKBP51 protein with an online tool (http://bioinfo.bjmu.edu.cn/lncpro/).
F. Schematic diagram of full length PCAT1 and PCAT1 truncated mutant (Δ1001-1400bp) (PCAT1-MUT).
G. PCAT1 truncated mutant (PCAT1-MUT) (Δ1001-1400bp) were created and transfected into LNCaP-AI cell line, and expression of PCAT1 and PCAT1-MUT were detected by RT-PCR (upper). AKT signaling and its downstream targets (phosphorylated 4E-BP1 and phosphorylated Erk1/2), NF-κB signaling and its downstream gene, c-Myc, were determined by Immunoblot in PCAT1-MUT overexpressed LNCaP-AI cells (lower). p-4E-BP1 (Thr37/46), phosphorylated 4E-BP1; p-Erk1/2 (Thr202/Thr204), phosphorylated Erk1/2.
H. Schematic diagram of GST-tagged full length FKBP51 (GST-FKBP51-WT) and GST-tagged FKBP51 truncated mutant (GST-FKBP51-MUT) (Δ251-390AA).
I. The ability of LNCaP-AI cells to form colonies were determined by colony assays after PCAT1 knockdown in the absence of androgen. The PCAT1-knockdown efficiency was presented in Figure 5a. Representative images are shown.
J. qRT-PCR detection of PCAT1 level in PCAT1-overexpressed cells combined with FKBP51-knockdown (or FKBP51-shSCR), normalized by the level of GAPDH. The efficiency of PCAT1 overexpression was shown in Figure 2D and efficiency of FKBP51-knockdown was presented in Figure 5H.

Table S1 and **Table S2:** Survival data of PCa patients in Genetic Amplification of PCAT1 cohort and Deep Deletion and without Genetic Alteration of PCAT1 cohort in TCGA dataset retrieved from cBioPortal.

Table S3: Expression of lncRNA PCAT1 in ADPC patients (n=498) retrieved from TCGA dataset and expression of lncRNA PCAT1 in CRPC patients (n=118) retrieved from SU2C/PCF Dream Team.

Table S4: RNA-seq data of decreased key genes regulated positively by AKT or NF-κB signal pathways in PCAT1 depleted LNCaP-AI cells ($p<0.05$).

Table S5 Possible lncRNAs that may interact with FKBP51 proteins and upregulated (Fold Change>2.0-fold, $p<0.01$) lncRNAs in the Arraystar Human LncRNA Microarray V3.0 data.

Figure S1.

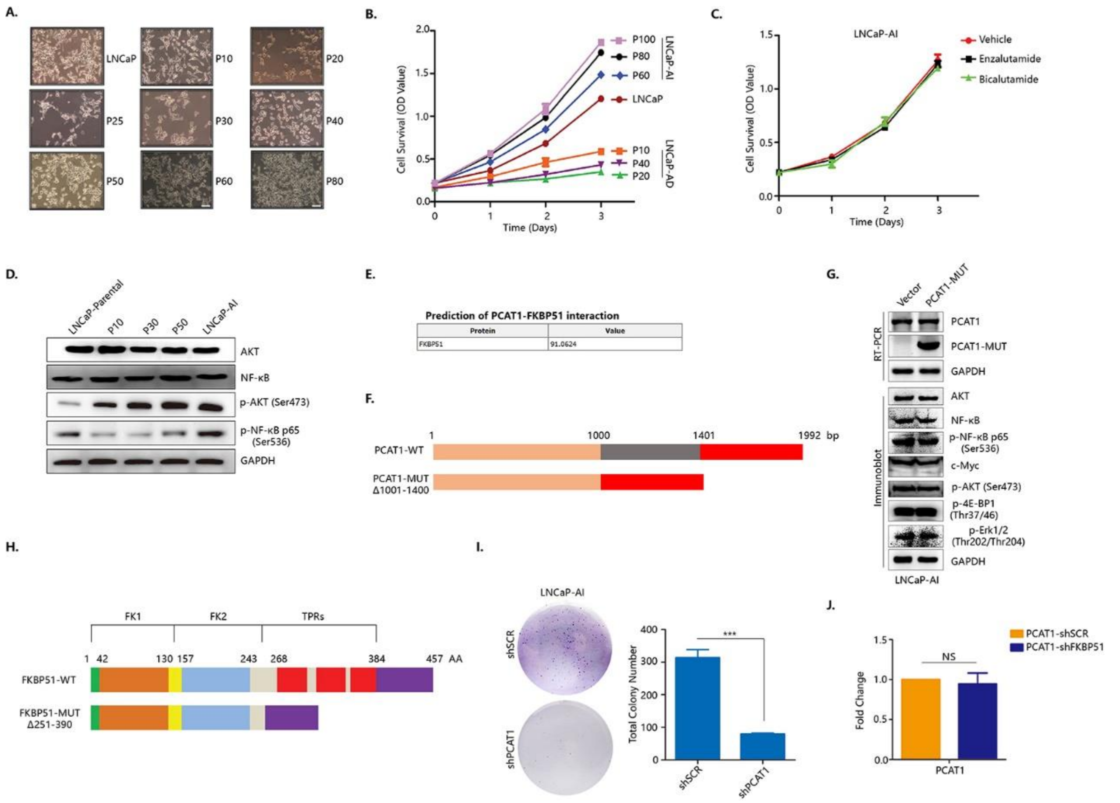
A.
LNCaP
P10
P20
P25
P30
P40
P50
P60
P80
B.
Cell Survival (OD Value)
Time (Days)
P100
P80
P60
LNCaP
P10
P40
P20
LNCaP-AI
LNCaP-AD
C.
LNCaP-AI
Vehicle
Enzalutamide
Bicalutamide
Cell Survival (OD Value)
Time (Days)
D.
LNCaP-Parental
P10
P30
P50
LNCaP-AI
AKT
NF-κB
p-AKT (Ser473)
p-NF-κB p65 (Ser536)
GAPDH
E.
Prediction of PCAT1-FKBP51 interaction
Protein
Value
FKBP51
91.0624
F.
1
1000
1401
1992 bp
PCAT1-WT
PCAT1-MUT Δ1001-1400
G.
Vector
PCAT1-MUT
RT-PCR
PCAT1
PCAT1-MUT
GAPDH
Immunoblot
AKT
NF-κB
p-NF-κB p65 (Ser536)
c-Myc
p-AKT (Ser473)
p-4E-BP1 (Thr37/46)
p-Erk1/2 (Thr202/Thr204)
GAPDH
LNCaP-AI
H.
FK1
FK2
TPRs
1 42 130 157 243 268 384 457 AA
FKBP51-WT
FKBP51-MUT Δ251-390
I.
LNCaP-AI
shSCR
shPCAT1
Total Colony Number
***
shSCR
shPCAT1
J.
PCAT1-shSCR
PCAT1-shFKBP51
NS
Fold Change
PCAT1

Table S1

Disease Free Survival Kaplan-Meier Estimate

Cases with Genetic Amplification of PCAT1

Case ID	Status	Time (months)
TCGA-CH-5739	censored	22.04
TCGA-CH-5753	censored	1.02
TCGA-CH-5754	censored	2.04
TCGA-CH-5767	censored	15.05
TCGA-EJ-5507	censored	47.86
TCGA-EJ-5527	censored	58.41
TCGA-EJ-5532	censored	60.05
TCGA-EJ-8469	relapsed	63.24
TCGA-EJ-A46G	censored	21.94
TCGA-FC-A4JI	censored	28.81
TCGA-G9-6373	censored	26.64
TCGA-G9-A9S0	relapsed	21.35
TCGA-HC-8265	censored	15.87
TCGA-HC-8266	censored	15.54
TCGA-HI-7168	relapsed	82.29
TCGA-J4-AATZ	relapsed	2.6
TCGA-J9-A8CL	relapsed	4.34
TCGA-J9-A8CM	relapsed	11.3
TCGA-KC-A4BL	relapsed	6.34
TCGA-KK-A59Y	censored	64.65
TCGA-KK-A7AP	censored	6.44
TCGA-KK-A7AQ	relapsed	39.98
TCGA-KK-A7AW	censored	34.63
TCGA-KK-A7AZ	censored	49.9
TCGA-KK-A7B4	relapsed	31.64
TCGA-KK-A8IA	censored	63.44
TCGA-KK-A8IF	relapsed	21.29
TCGA-V1-A905	relapsed	4.07
TCGA-V1-A9Z7	censored	28.71
TCGA-V1-A9ZK	censored	45.43
TCGA-XJ-A9DX	censored	31.96
TCGA-XK-AAIV	censored	37.22
TCGA-YJ-A8SW	censored	4.86
TCGA-ZG-A9KY	censored	4.27
TCGA-ZG-A9ND	censored	13.47

Cases with Genetic Deep Deletion and without

Case ID	Status	Time (months)
TCGA-HC-A4ZV	censored	0.76
TCGA-TK-A8OK	censored	0.89
TCGA-CH-5761	censored	0.92
TCGA-CH-5748	censored	1.02
TCGA-CH-5740	censored	1.02
TCGA-CH-5764	censored	1.02
TCGA-CH-5766	censored	1.02
TCGA-HC-A6HX	censored	1.25
TCGA-HC-A6AS	censored	1.45

TCGA-HC-8264	censored	1.58
TCGA-HC-A6AN	censored	1.61
TCGA-ZG-A9L9	relapsed	1.68
TCGA-HC-A631	censored	1.77
TCGA-J9-A52B	relapsed	1.87
TCGA-CH-5744	censored	1.97
TCGA-HC-A632	censored	2
TCGA-CH-5769	censored	2.04
TCGA-HC-A6AL	censored	2.2
TCGA-HC-A6AP	censored	2.33
TCGA-EJ-A65F	relapsed	2.46
TCGA-KK-A8IB	censored	2.73
TCGA-XJ-A9DQ	censored	2.99
TCGA-CH-5737	censored	2.99
TCGA-KC-A7F5	censored	2.99
TCGA-CH-5745	censored	2.99
TCGA-ZG-A8QW	censored	3.09
TCGA-QU-A6IL	censored	3.19
TCGA-V1-A9OL	relapsed	3.45
TCGA-HC-A6AQ	censored	3.48
TCGA-H9-A6BY	censored	3.68
TCGA-2A-A8W1	censored	3.68
TCGA-EJ-7793	censored	3.75
TCGA-ZG-A9LN	censored	3.78
TCGA-EJ-A8FP	relapsed	3.84
TCGA-V1-A9O5	relapsed	4.07
TCGA-EJ-A7NM	censored	4.2
TCGA-EJ-AB20	censored	4.3
TCGA-XK-AAJR	relapsed	4.3
TCGA-ZG-A9NI	censored	4.34
TCGA-HC-A6HY	censored	4.4
TCGA-EJ-A8FU	censored	4.53
TCGA-EJ-AB27	censored	4.76
TCGA-HC-A76X	censored	4.93
TCGA-YL-A8SC	relapsed	4.99
TCGA-HC-7213	relapsed	5.58
TCGA-MG-AAMC	censored	5.68
TCGA-J9-A52C	censored	5.85
TCGA-ZG-A9L2	relapsed	5.91
TCGA-J4-A67T	censored	6.01
TCGA-H9-7775	censored	6.08
TCGA-VN-A88M	censored	6.21
TCGA-EJ-7330	censored	6.27
TCGA-EJ-8472	relapsed	6.44
TCGA-EJ-A7NJ	censored	6.47
TCGA-EJ-A7NN	relapsed	6.47
TCGA-2A-A8W3	relapsed	6.5
TCGA-V1-A8X3	censored	6.64
TCGA-KK-A5A1	relapsed	6.67
TCGA-KK-A7AU	relapsed	6.8
TCGA-J9-A52D	censored	6.96
TCGA-CH-5738	censored	6.96
TCGA-HC-A76W	censored	7
TCGA-EJ-A46F	relapsed	7.06

TCGA-EJ-A8FS	relapsed	7.1
TCGA-HC-A9TE	relapsed	7.1
TCGA-EJ-A65M	censored	7.56
TCGA-EJ-A7NF	censored	8.21
TCGA-KC-A7F6	censored	8.64
TCGA-EJ-5524	relapsed	8.71
TCGA-VN-A88I	censored	8.84
TCGA-KC-A7FD	censored	8.87
TCGA-YL-A8SR	censored	8.97
TCGA-XJ-A9DK	censored	9.03
TCGA-EJ-A8FO	censored	9.26
TCGA-HC-A8CY	censored	9.53
TCGA-V1-A9OT	relapsed	9.59
TCGA-KK-A7B3	relapsed	9.66
TCGA-CH-5789	censored	9.99
TCGA-KK-A8IJ	relapsed	10.09
TCGA-ZG-A8QZ	censored	10.12
TCGA-J9-A8CK	censored	10.55
TCGA-J9-A52E	censored	10.61
TCGA-J4-A83K	censored	10.78
TCGA-EJ-A8FN	censored	10.81
TCGA-YL-A8SQ	relapsed	10.81
TCGA-ZG-A9N3	censored	11.47
TCGA-4L-AA1F	censored	11.5
TCGA-HC-A9TH	relapsed	11.53
TCGA-KC-A7FE	censored	11.56
TCGA-EJ-A6RA	relapsed	11.6
TCGA-ZG-A9L0	censored	11.66
TCGA-CH-5751	relapsed	11.99
TCGA-CH-5763	censored	11.99
TCGA-ZG-A9LY	censored	12.16
TCGA-V1-A9OQ	censored	12.19
TCGA-XA-A8JR	censored	12.35
TCGA-ZG-A9LM	censored	12.42
TCGA-EJ-7318	relapsed	12.48
TCGA-HC-7079	relapsed	12.48
TCGA-M7-A720	censored	12.61
TCGA-J9-A8CP	censored	12.68
TCGA-EJ-A65D	censored	12.91
TCGA-CH-5741	censored	12.98
TCGA-CH-5750	censored	13.01
TCGA-CH-5771	censored	13.01
TCGA-M7-A721	censored	13.14
TCGA-ZG-A8QY	censored	13.27
TCGA-EJ-5497	censored	13.3
TCGA-EJ-A65E	censored	13.7
TCGA-HC-7738	relapsed	13.8
TCGA-CH-5743	relapsed	13.96
TCGA-XK-AAIW	relapsed	14.03
TCGA-YL-A9WH	censored	14.03
TCGA-EJ-A7NG	censored	14.19
TCGA-J4-A83N	relapsed	14.22
TCGA-HC-7209	censored	14.45
TCGA-J4-A67N	relapsed	14.52

TCGA-ZG-A8QX	censored	14.52
TCGA-V1-A8ML	censored	14.72
TCGA-WW-A8ZI	censored	14.75
TCGA-EJ-A65J	censored	14.82
TCGA-ZG-A9MC	censored	14.95
TCGA-FC-7961	censored	15.41
TCGA-V1-A8WS	censored	15.57
TCGA-EJ-A7NH	censored	15.57
TCGA-EJ-A7NK	censored	15.64
TCGA-VN-A88O	censored	15.8
TCGA-CH-5772	censored	15.97
TCGA-M7-A71Y	censored	16.2
TCGA-VN-A943	censored	16.29
TCGA-V1-A8MK	censored	16.69
TCGA-J4-A6M7	censored	16.82
TCGA-YL-A8SA	censored	16.82
TCGA-VN-A88R	relapsed	16.82
TCGA-ZG-A9LS	censored	16.95
TCGA-J4-A83M	relapsed	17.12
TCGA-HC-7750	censored	17.15
TCGA-FC-A66V	censored	17.21
TCGA-EJ-A46E	censored	17.21
TCGA-TP-A8TT	censored	17.28
TCGA-Y6-A9XI	censored	17.28
TCGA-HC-A6AO	censored	17.41
TCGA-HC-7744	censored	17.44
TCGA-V1-A9Z9	censored	17.74
TCGA-HC-7819	censored	17.84
TCGA-HC-8261	censored	17.94
TCGA-EJ-5525	relapsed	17.94
TCGA-ZG-A9M4	censored	17.97
TCGA-SU-A7E7	censored	18.1
TCGA-J4-AATV	censored	18.17
TCGA-M7-A722	relapsed	18.36
TCGA-M7-A725	censored	18.82
TCGA-HC-A8D1	censored	18.82
TCGA-ZG-A9LB	censored	19.15
TCGA-HC-8213	censored	19.35
TCGA-EJ-5496	censored	19.55
TCGA-KC-A7FA	censored	19.65
TCGA-HC-7075	censored	19.74
TCGA-KK-A7B0	relapsed	19.91
TCGA-KC-A4BO	censored	19.94
TCGA-TP-A8TV	censored	20.04
TCGA-J4-8198	censored	20.17
TCGA-2A-AAYU	censored	20.2
TCGA-FC-A8O0	censored	20.24
TCGA-J4-A6G3	relapsed	20.3
TCGA-EJ-A46D	censored	20.57
TCGA-V1-A9OA	censored	20.89
TCGA-M7-A71Z	censored	21.12
TCGA-EJ-A46B	censored	21.58
TCGA-VP-A87H	censored	21.78
TCGA-ZG-A9L6	relapsed	21.81

TCGA-KC-A7F3	censored	21.85
TCGA-EJ-A65G	censored	21.88
TCGA-KK-A8I6	censored	21.94
TCGA-EJ-A46I	censored	21.94
TCGA-2A-A8VV	censored	22.04
TCGA-YL-A8S8	relapsed	22.31
TCGA-HC-8262	censored	22.31
TCGA-HC-A48F	censored	22.34
TCGA-FC-A50B	censored	22.34
TCGA-HC-8216	censored	22.4
TCGA-J4-A83I	censored	22.5
TCGA-V1-A8WV	censored	22.54
TCGA-HC-8260	censored	22.54
TCGA-KK-A7B2	relapsed	22.73
TCGA-ZG-A9LZ	censored	22.73
TCGA-J4-A83J	censored	22.86
TCGA-VP-A87K	relapsed	22.9
TCGA-CH-5765	censored	23
TCGA-J4-A67S	relapsed	23.26
TCGA-EJ-A65B	censored	23.32
TCGA-HC-7740	censored	23.55
TCGA-J4-A83L	censored	23.65
TCGA-HC-7745	censored	23.69
TCGA-HC-7749	censored	23.78
TCGA-EJ-A46H	censored	23.78
TCGA-HC-8257	censored	23.85
TCGA-G9-A9S7	censored	23.92
TCGA-CH-5746	censored	24.01
TCGA-CH-5768	censored	24.01
TCGA-YL-A9WL	relapsed	24.31
TCGA-EJ-7331	censored	24.38
TCGA-VN-A88L	censored	24.51
TCGA-HC-7820	censored	24.54
TCGA-HC-7747	censored	24.57
TCGA-YL-A8SJ	relapsed	24.7
TCGA-M7-A723	censored	25.07
TCGA-G9-6384	censored	25.13
TCGA-EJ-8474	censored	25.16
TCGA-HC-7232	relapsed	25.16
TCGA-VN-A88N	censored	25.16
TCGA-J4-A6G1	censored	25.26
TCGA-J4-A67L	censored	25.3
TCGA-XQ-A8TB	censored	25.3
TCGA-EJ-7317	censored	25.33
TCGA-HC-A8D0	censored	25.46
TCGA-VN-A88K	censored	25.49
TCGA-EJ-7784	censored	25.66
TCGA-HC-7752	censored	25.69
TCGA-EJ-7783	censored	25.85
TCGA-FC-A6HD	censored	25.92
TCGA-HC-7748	censored	26.02
TCGA-V1-A9O7	relapsed	26.91
TCGA-CH-5788	censored	26.97
TCGA-KK-A7AV	censored	27

TCGA-EJ-7321	censored	27.07
TCGA-J4-AAU2	censored	27.1
TCGA-V1-A9Z8	censored	27.17
TCGA-G9-6385	censored	27.27
TCGA-J4-A670	censored	27.56
TCGA-EJ-7328	censored	27.66
TCGA-HC-8258	censored	27.83
TCGA-G9-7519	censored	27.89
TCGA-G9-7523	censored	28.15
TCGA-V1-A8MG	censored	28.15
TCGA-EJ-7788	censored	28.22
TCGA-HC-8256	censored	28.25
TCGA-FC-7708	censored	28.38
TCGA-HC-7210	censored	28.52
TCGA-HC-7212	censored	28.58
TCGA-EJ-A6RC	censored	28.68
TCGA-CH-5794	censored	28.98
TCGA-M7-A724	censored	29.2
TCGA-HC-7080	relapsed	29.37
TCGA-G9-A9S4	censored	29.76
TCGA-ZG-A9L5	censored	29.83
TCGA-J4-A67R	censored	29.89
TCGA-EJ-5505	censored	30.29
TCGA-EJ-7327	censored	30.32
TCGA-V1-A8WN	censored	30.35
TCGA-HC-8259	censored	30.35
TCGA-KK-A6E7	relapsed	30.39
TCGA-HC-7817	censored	30.49
TCGA-XK-AAJA	censored	30.55
TCGA-HC-7818	censored	30.81
TCGA-HC-7742	censored	30.81
TCGA-HC-7231	censored	30.85
TCGA-KK-A8I9	relapsed	30.88
TCGA-KK-A6E0	relapsed	30.91
TCGA-J4-A67M	censored	30.91
TCGA-H9-A6BX	censored	30.91
TCGA-G9-7521	censored	30.95
TCGA-CH-5752	censored	30.98
TCGA-HC-7821	censored	31.41
TCGA-G9-6377	censored	31.47
TCGA-KK-A8I8	censored	31.77
TCGA-XK-AAIR	censored	31.93
TCGA-CH-5790	censored	32
TCGA-CH-5791	relapsed	32
TCGA-J4-A67Q	censored	32.03
TCGA-YL-A9WY	relapsed	32.03
TCGA-X4-A8KS	censored	32.16
TCGA-XK-AAK1	censored	32.16
TCGA-EJ-7797	censored	32.29
TCGA-YL-A8SL	censored	32.42
TCGA-V1-A8MM	relapsed	32.52
TCGA-Y6-A8TL	censored	32.59
TCGA-G9-7525	censored	32.65
TCGA-HC-7230	censored	32.88

TCGA-J4-A67K	censored	32.98
TCGA-YL-A8S9	relapsed	33.05
TCGA-EJ-7315	censored	33.11
TCGA-YL-A9WK	relapsed	33.15
TCGA-KC-A4BR	relapsed	33.57
TCGA-KK-A8IC	relapsed	34.82
TCGA-KK-A7B1	censored	34.92
TCGA-V1-A9OY	censored	35.05
TCGA-YL-A8HO	relapsed	35.09
TCGA-EJ-7325	censored	35.15
TCGA-EJ-7781	censored	35.25
TCGA-V1-A8WW	censored	35.41
TCGA-G9-7522	censored	35.41
TCGA-HC-7737	censored	35.81
TCGA-EJ-7786	censored	36.01
TCGA-EJ-7312	censored	36.1
TCGA-HC-7736	censored	36.24
TCGA-EJ-7789	censored	36.3
TCGA-ZG-A9L4	censored	36.4
TCGA-EJ-7791	censored	36.73
TCGA-KK-A7AY	relapsed	36.93
TCGA-XJ-A83F	censored	37.02
TCGA-V1-A9OX	censored	37.25
TCGA-HC-7081	censored	37.32
TCGA-EJ-7314	censored	37.78
TCGA-G9-6378	censored	37.94
TCGA-G9-6370	censored	37.98
TCGA-EJ-8470	censored	38.07
TCGA-EJ-7782	censored	38.34
TCGA-EJ-5502	censored	38.47
TCGA-EJ-7785	censored	38.67
TCGA-V1-A9OF	censored	38.9
TCGA-G9-7510	censored	38.93
TCGA-VP-A87D	relapsed	39.22
TCGA-G9-6364	censored	39.36
TCGA-G9-6369	censored	39.91
TCGA-J4-8200	censored	40.01
TCGA-HC-7211	censored	40.11
TCGA-G9-6367	censored	40.14
TCGA-G9-6371	censored	40.28
TCGA-J9-A8CN	censored	40.64
TCGA-QU-A6IM	censored	40.97
TCGA-XK-AAJP	censored	41.36
TCGA-VN-A88P	censored	41.39
TCGA-XK-AAJ3	censored	41.46
TCGA-G9-6329	censored	41.59
TCGA-2A-AAYO	censored	41.79
TCGA-EJ-5531	censored	41.82
TCGA-XJ-A83H	censored	42.05
TCGA-G9-6332	relapsed	42.21
TCGA-ZG-A9L1	censored	42.77
TCGA-EJ-7794	censored	42.9
TCGA-KC-A4BV	relapsed	43.63
TCGA-CH-5762	censored	43.99

TCGA-G9-6498	relapsed	44.09
TCGA-HC-7233	censored	44.51
TCGA-V1-A9ZR	censored	44.71
TCGA-G9-6365	censored	44.78
TCGA-2A-AAYF	censored	44.81
TCGA-VP-AA1N	censored	44.84
TCGA-EJ-5501	censored	44.84
TCGA-VN-A88Q	censored	44.88
TCGA-YL-A8SF	censored	45.11
TCGA-2A-A8VT	censored	45.11
TCGA-YL-A8HK	relapsed	45.2
TCGA-G9-6363	censored	45.27
TCGA-2A-A8VX	censored	45.27
TCGA-EJ-5506	censored	45.37
TCGA-X4-A8KQ	censored	45.43
TCGA-YL-A8SB	relapsed	45.47
TCGA-EJ-5503	censored	45.7
TCGA-EJ-7792	censored	45.86
TCGA-YL-A8SK	censored	46.25
TCGA-G9-6361	censored	46.48
TCGA-EJ-5499	censored	46.48
TCGA-YL-A8SI	relapsed	46.75
TCGA-EJ-5504	censored	46.88
TCGA-G9-6356	censored	47.11
TCGA-XK-AAJT	censored	47.11
TCGA-G9-6362	censored	47.4
TCGA-V1-A8MF	censored	47.6
TCGA-XJ-A9DI	censored	48
TCGA-XJ-A83G	censored	48
TCGA-EJ-5509	censored	48.09
TCGA-YL-A8HM	relapsed	48.39
TCGA-EJ-5511	censored	48.49
TCGA-EJ-5494	censored	48.52
TCGA-YL-A9WX	relapsed	49.47
TCGA-EJ-5542	censored	49.7
TCGA-G9-6348	censored	49.77
TCGA-V1-A9ZG	censored	49.84
TCGA-G9-6353	censored	50.66
TCGA-G9-6499	censored	50.69
TCGA-YL-A8HL	censored	51.41
TCGA-XK-AAJU	censored	51.77
TCGA-G9-7509	censored	52.89
TCGA-YL-A8HJ	censored	52.92
TCGA-YL-A9WI	censored	53.32
TCGA-G9-6339	relapsed	53.68
TCGA-V1-A9ZI	censored	53.88
TCGA-VP-A87C	censored	55.52
TCGA-G9-6342	censored	55.72
TCGA-2A-A8VO	censored	55.88
TCGA-EJ-5498	censored	56.5
TCGA-G9-6496	censored	56.7
TCGA-EJ-5512	censored	56.93
TCGA-KK-A8I7	relapsed	57.69
TCGA-EJ-5495	censored	57.82

TCGA-KK-A6E8	censored	58.15
TCGA-G9-6494	censored	58.18
TCGA-VP-A87J	censored	58.28
TCGA-G9-6379	censored	58.41
TCGA-V1-A8MJ	censored	58.84
TCGA-KC-A4BN	censored	59.63
TCGA-EJ-5514	censored	60.09
TCGA-EJ-5530	censored	60.18
TCGA-EJ-5515	censored	60.18
TCGA-KK-A8II	relapsed	60.91
TCGA-V1-A8MU	censored	61.63
TCGA-EJ-5510	censored	61.99
TCGA-EJ-5516	censored	62.06
TCGA-EJ-5517	censored	62.06
TCGA-EJ-5526	relapsed	62.71
TCGA-KK-A8IM	censored	62.91
TCGA-G9-6366	censored	63.96
TCGA-V1-A8WL	censored	63.99
TCGA-EJ-5519	censored	64.45
TCGA-EJ-5508	censored	64.55
TCGA-HC-7078	censored	65.21
TCGA-KK-A8IH	censored	66.13
TCGA-G9-6338	censored	66.62
TCGA-YL-A8SP	relapsed	66.89
TCGA-VP-A87E	censored	66.92
TCGA-G9-6351	censored	67.28
TCGA-KK-A8IK	censored	67.41
TCGA-KK-A6E3	censored	67.54
TCGA-G9-6354	censored	67.87
TCGA-G9-6336	censored	67.94
TCGA-KK-A6E5	censored	68.1
TCGA-EJ-5522	censored	68.3
TCGA-G9-6347	censored	68.63
TCGA-EJ-5518	relapsed	69.12
TCGA-KK-A8ID	censored	69.22
TCGA-HC-7077	censored	69.48
TCGA-KK-A8I4	relapsed	71.02
TCGA-KK-A6E1	censored	72.21
TCGA-KK-A59X	relapsed	73.36
TCGA-EJ-5521	censored	74.87
TCGA-VP-A875	censored	75.85
TCGA-EJ-8468	censored	77.1
TCGA-VP-A878	relapsed	77.33
TCGA-V1-A9OH	censored	78.94
TCGA-G9-6343	censored	80.49
TCGA-G9-6333	censored	80.98
TCGA-VP-A87B	relapsed	81.24
TCGA-KK-A8IG	censored	82.29
TCGA-HI-7170	censored	82.85
TCGA-EJ-7218	censored	83.51
TCGA-EJ-7123	censored	84.49
TCGA-QU-A6IP	censored	86.07
TCGA-KK-A59Z	censored	86.33
TCGA-HI-7169	censored	88.17

TCGA-EJ-7115	censored	88.27
TCGA-EJ-7125	censored	93.63
TCGA-V1-A909	censored	94.28
TCGA-KK-A8I5	censored	101.71
TCGA-VP-A876	censored	109.49
TCGA-KK-A59V	censored	113.01
TCGA-KK-A6E6	censored	113.24
TCGA-YL-A8SO	censored	114.29
TCGA-VP-A872	censored	119.28
TCGA-QU-A6IO	censored	122.08
TCGA-QU-A6IN	censored	140.08
TCGA-KK-A6DY	censored	141.1
TCGA-KK-A6E2	censored	165.05
TCGA-YL-A9WJ	relapsed	48.49
TCGA-ZG-A9LU	censored	19.02
TCGA-CH-5792	censored	2.99

Table S2

Overall Survival Kaplan-Meier Estimate

Cases with Genetic Amplification of

Case ID	Status	Time (months)
TCGA-CH-5739	censored	22.04
TCGA-CH-5753	censored	1.02
TCGA-CH-5754	censored	2.04
TCGA-CH-5767	censored	15.05
TCGA-EJ-5507	censored	47.86
TCGA-EJ-5527	censored	58.41
TCGA-EJ-5532	censored	60.05
TCGA-EJ-8469	censored	76.35
TCGA-EJ-A46G	censored	21.94
TCGA-FC-A4JI	censored	28.81
TCGA-G9-6373	censored	26.64
TCGA-G9-A9S0	deceased	25.99
TCGA-HC-8265	censored	15.87
TCGA-HC-8266	censored	15.54
TCGA-HI-7168	censored	109.17
TCGA-HI-7171	deceased	43.66
TCGA-J4-AATZ	censored	13.53
TCGA-J9-A8CL	censored	13.3
TCGA-J9-A8CM	censored	15.21
TCGA-KC-A4BL	censored	30.68
TCGA-KK-A59Y	censored	64.65
TCGA-KK-A7AP	censored	6.44
TCGA-KK-A7AQ	censored	52.89
TCGA-KK-A7AW	censored	34.63
TCGA-KK-A7AZ	censored	49.9
TCGA-KK-A7B4	censored	32.42
TCGA-KK-A8IA	censored	63.44
TCGA-KK-A8IF	censored	26.41
TCGA-V1-A9O5	censored	63.47
TCGA-V1-A9Z7	censored	28.71
TCGA-V1-A9ZK	censored	45.43
TCGA-XJ-A9DX	censored	31.96
TCGA-XK-AAIV	censored	37.22
TCGA-XQ-A8TA	deceased	4.8
TCGA-YJ-A8SW	censored	4.86
TCGA-ZG-A9KY	censored	4.27
TCGA-ZG-A9ND	censored	13.47

Cases with Genetic Deep Deletion and

Case ID	Status	Time (months)
TCGA-HC-A4ZV	censored	0.76
TCGA-TK-A8OK	censored	0.89
TCGA-CH-5761	censored	0.92
TCGA-CH-5748	censored	1.02
TCGA-CH-5740	censored	1.02
TCGA-CH-5764	censored	1.02
TCGA-CH-5766	censored	1.02
TCGA-HC-A6HX	censored	1.25

TCGA-HC-A6AS	censored	1.45
TCGA-HC-8264	censored	1.58
TCGA-HC-A6AN	censored	1.61
TCGA-HC-A631	censored	1.77
TCGA-CH-5744	censored	1.97
TCGA-HC-A632	censored	2
TCGA-CH-5769	censored	2.04
TCGA-HC-A6AL	censored	2.2
TCGA-HC-A6AP	censored	2.33
TCGA-KK-A8IB	censored	2.73
TCGA-XJ-A9DQ	censored	2.99
TCGA-CH-5737	censored	2.99
TCGA-KC-A7F5	censored	2.99
TCGA-CH-5745	censored	2.99
TCGA-ZG-A8QW	censored	3.09
TCGA-QU-A6IL	censored	3.19
TCGA-HC-A6AQ	censored	3.48
TCGA-H9-A6BY	censored	3.68
TCGA-2A-A8W1	censored	3.68
TCGA-EJ-7793	censored	3.75
TCGA-ZG-A9LN	censored	3.78
TCGA-EJ-A7NM	censored	4.2
TCGA-EJ-AB20	censored	4.3
TCGA-ZG-A9NI	censored	4.34
TCGA-HC-A6HY	censored	4.4
TCGA-EJ-A8FU	censored	4.53
TCGA-EJ-AB27	censored	4.76
TCGA-HC-A76X	censored	4.93
TCGA-MG-AAMC	censored	5.68
TCGA-J9-A52C	censored	5.85
TCGA-J4-A67T	censored	6.01
TCGA-H9-7775	censored	6.08
TCGA-VN-A88M	censored	6.21
TCGA-EJ-7330	censored	6.27
TCGA-EJ-A7NJ	censored	6.47
TCGA-EJ-A7NN	censored	6.47
TCGA-V1-A8X3	censored	6.64
TCGA-J9-A52D	censored	6.96
TCGA-CH-5738	censored	6.96
TCGA-HC-A76W	censored	7
TCGA-EJ-A65M	censored	7.56
TCGA-EJ-A7NF	censored	8.21
TCGA-KC-A7F6	censored	8.64
TCGA-EJ-A8FS	censored	8.74
TCGA-VN-A88I	censored	8.84
TCGA-KC-A7FD	censored	8.87
TCGA-YL-A8SR	censored	8.97
TCGA-XJ-A9DK	censored	9.03
TCGA-EJ-A8FO	censored	9.26
TCGA-HC-A8CY	censored	9.53
TCGA-EJ-A8FP	censored	9.82
TCGA-CH-5789	censored	9.99
TCGA-ZG-A8QZ	censored	10.12
TCGA-J9-A8CK	censored	10.55

TCGA-J9-A52E	censored	10.61
TCGA-J4-A83K	censored	10.78
TCGA-EJ-A8FN	censored	10.81
TCGA-ZG-A9N3	censored	11.47
TCGA-4L-AA1F	censored	11.5
TCGA-KC-A7FE	censored	11.56
TCGA-EJ-A6RA	censored	11.6
TCGA-ZG-A9L0	censored	11.66
TCGA-CH-5763	censored	11.99
TCGA-ZG-A9LY	censored	12.16
TCGA-V1-A9OQ	censored	12.19
TCGA-XA-A8JR	censored	12.35
TCGA-ZG-A9LM	censored	12.42
TCGA-M7-A720	censored	12.61
TCGA-J9-A8CP	censored	12.68
TCGA-EJ-A65D	censored	12.91
TCGA-CH-5741	censored	12.98
TCGA-CH-5750	censored	13.01
TCGA-CH-5771	censored	13.01
TCGA-M7-A721	censored	13.14
TCGA-ZG-A8QY	censored	13.27
TCGA-EJ-5497	censored	13.3
TCGA-EJ-A65E	censored	13.7
TCGA-J9-A52B	censored	13.86
TCGA-CH-5743	censored	13.96
TCGA-YL-A9WH	censored	14.03
TCGA-EJ-A7NG	censored	14.19
TCGA-HC-7209	censored	14.45
TCGA-ZG-A8QX	censored	14.52
TCGA-V1-A8ML	censored	14.72
TCGA-WW-A8ZI	censored	14.75
TCGA-EJ-A65J	censored	14.82
TCGA-ZG-A9MC	censored	14.95
TCGA-FC-7961	censored	15.41
TCGA-V1-A8WS	censored	15.57
TCGA-EJ-A7NH	censored	15.57
TCGA-EJ-A7NK	censored	15.64
TCGA-V1-A9OT	censored	15.64
TCGA-VN-A88O	censored	15.8
TCGA-CH-5772	censored	15.97
TCGA-M7-A71Y	censored	16.2
TCGA-VN-A943	censored	16.29
TCGA-V1-A8MK	censored	16.69
TCGA-J4-A6M7	censored	16.82
TCGA-YL-A8SA	censored	16.82
TCGA-ZG-A9LS	censored	16.95
TCGA-HC-7750	censored	17.15
TCGA-FC-A66V	censored	17.21
TCGA-EJ-A46E	censored	17.21
TCGA-TP-A8TT	censored	17.28
TCGA-Y6-A9XI	censored	17.28
TCGA-HC-A6AO	censored	17.41
TCGA-HC-7744	censored	17.44
TCGA-V1-A9Z9	censored	17.74

TCGA-HC-7819	censored	17.84
TCGA-J4-A83M	censored	17.84
TCGA-HC-8261	censored	17.94
TCGA-ZG-A9M4	censored	17.97
TCGA-SU-A7E7	censored	18.1
TCGA-J4-AATV	censored	18.17
TCGA-EJ-A65F	censored	18.4
TCGA-M7-A725	censored	18.82
TCGA-HC-A8D1	censored	18.82
TCGA-ZG-A9LB	censored	19.15
TCGA-HC-A9TE	censored	19.28
TCGA-HC-8213	censored	19.35
TCGA-EJ-5496	censored	19.55
TCGA-KC-A7FA	censored	19.65
TCGA-HC-7075	censored	19.74
TCGA-KC-A4BO	censored	19.94
TCGA-TP-A8TV	censored	20.04
TCGA-J4-8198	censored	20.17
TCGA-2A-AAYU	censored	20.2
TCGA-FC-A8O0	censored	20.24
TCGA-ZG-A9L9	censored	20.27
TCGA-EJ-A46D	censored	20.57
TCGA-KK-A8IL	deceased	20.63
TCGA-V1-A9OA	censored	20.89
TCGA-M7-A71Z	censored	21.12
TCGA-EJ-A46B	censored	21.58
TCGA-VP-A87H	censored	21.78
TCGA-KC-A7F3	censored	21.85
TCGA-EJ-A65G	censored	21.88
TCGA-KK-A8I6	censored	21.94
TCGA-EJ-A46I	censored	21.94
TCGA-2A-A8VV	censored	22.04
TCGA-HC-8262	censored	22.31
TCGA-HC-A48F	censored	22.34
TCGA-FC-A5OB	censored	22.34
TCGA-HC-8216	censored	22.4
TCGA-J4-A83I	censored	22.5
TCGA-V1-A8WV	censored	22.54
TCGA-HC-8260	censored	22.54
TCGA-ZG-A9L6	censored	22.57
TCGA-ZG-A9LZ	censored	22.73
TCGA-J4-A83J	censored	22.86
TCGA-CH-5765	censored	23
TCGA-EJ-A65B	censored	23.32
TCGA-HC-7740	censored	23.55
TCGA-J4-A83L	censored	23.65
TCGA-HC-7745	censored	23.69
TCGA-HC-7749	censored	23.78
TCGA-EJ-A46H	censored	23.78
TCGA-HC-8257	censored	23.85
TCGA-G9-A9S7	censored	23.92
TCGA-VP-A879	deceased	23.92
TCGA-CH-5746	censored	24.01
TCGA-CH-5768	censored	24.01

TCGA-YL-A9WL	censored	24.31
TCGA-EJ-7331	censored	24.38
TCGA-VN-A88L	censored	24.51
TCGA-HC-7820	censored	24.54
TCGA-HC-7747	censored	24.57
TCGA-EJ-8472	censored	24.84
TCGA-M7-A723	censored	25.07
TCGA-G9-6384	censored	25.13
TCGA-EJ-8474	censored	25.16
TCGA-VN-A88N	censored	25.16
TCGA-J4-A6G1	censored	25.26
TCGA-J4-A67L	censored	25.3
TCGA-XQ-A8TB	censored	25.3
TCGA-EJ-7317	censored	25.33
TCGA-HC-A8D0	censored	25.46
TCGA-VN-A88K	censored	25.49
TCGA-EJ-7784	censored	25.66
TCGA-HC-7752	censored	25.69
TCGA-EJ-A46F	censored	25.72
TCGA-VN-A88R	censored	25.76
TCGA-EJ-7783	censored	25.85
TCGA-FC-A6HD	censored	25.92
TCGA-HC-7748	censored	26.02
TCGA-CH-5788	censored	26.97
TCGA-KK-A7AV	censored	27
TCGA-EJ-7321	censored	27.07
TCGA-J4-AAU2	censored	27.1
TCGA-V1-A9Z8	censored	27.17
TCGA-G9-6385	censored	27.27
TCGA-J4-A67S	censored	27.37
TCGA-J4-A67O	censored	27.56
TCGA-EJ-7328	censored	27.66
TCGA-HC-A9TH	censored	27.73
TCGA-HC-8258	censored	27.83
TCGA-J4-A6G3	censored	27.89
TCGA-G9-7519	censored	27.89
TCGA-G9-7523	censored	28.15
TCGA-V1-A8MG	censored	28.15
TCGA-EJ-7788	censored	28.22
TCGA-HC-8256	censored	28.25
TCGA-2A-A8W3	censored	28.35
TCGA-FC-7708	censored	28.38
TCGA-HC-7210	censored	28.52
TCGA-HC-7212	censored	28.58
TCGA-EJ-A6RC	censored	28.68
TCGA-YL-A8SH	deceased	28.75
TCGA-CH-5794	censored	28.98
TCGA-M7-A724	censored	29.2
TCGA-KK-A7B3	censored	29.53
TCGA-G9-A9S4	censored	29.76
TCGA-ZG-A9L5	censored	29.83
TCGA-J4-A67R	censored	29.89
TCGA-EJ-5505	censored	30.29
TCGA-EJ-7327	censored	30.32

TCGA-V1-A8WN	censored	30.35
TCGA-HC-8259	censored	30.35
TCGA-HC-7817	censored	30.49
TCGA-XK-AAJA	censored	30.55
TCGA-J4-A67N	censored	30.78
TCGA-HC-7818	censored	30.81
TCGA-HC-7742	censored	30.81
TCGA-HC-7231	censored	30.85
TCGA-J4-A67M	censored	30.91
TCGA-H9-A6BX	censored	30.91
TCGA-G9-7521	censored	30.95
TCGA-CH-5752	censored	30.98
TCGA-HC-7821	censored	31.41
TCGA-G9-6377	censored	31.47
TCGA-HC-7738	censored	31.67
TCGA-KK-A8I8	censored	31.77
TCGA-XK-AAIR	censored	31.93
TCGA-CH-5790	censored	32
TCGA-J4-A67Q	censored	32.03
TCGA-X4-A8KS	censored	32.16
TCGA-XK-AAK1	censored	32.16
TCGA-EJ-7797	censored	32.29
TCGA-YL-A8SL	censored	32.42
TCGA-J4-A83N	censored	32.59
TCGA-Y6-A8TL	censored	32.59
TCGA-G9-7525	censored	32.65
TCGA-KK-A8I9	censored	32.88
TCGA-HC-7230	censored	32.88
TCGA-J4-A67K	censored	32.98
TCGA-CH-5791	censored	32.98
TCGA-EJ-7315	censored	33.11
TCGA-EJ-7318	censored	33.8
TCGA-M7-A722	censored	34.82
TCGA-KK-A7B1	censored	34.92
TCGA-CH-5751	censored	34.99
TCGA-V1-A9OY	censored	35.05
TCGA-EJ-7325	censored	35.15
TCGA-EJ-7781	censored	35.25
TCGA-V1-A8WW	censored	35.41
TCGA-G9-7522	censored	35.41
TCGA-HC-7737	censored	35.81
TCGA-EJ-7786	censored	36.01
TCGA-YL-A9WY	censored	36.07
TCGA-KK-A7B2	censored	36.1
TCGA-EJ-7312	censored	36.1
TCGA-HC-7736	censored	36.24
TCGA-EJ-7789	censored	36.3
TCGA-HC-7080	censored	36.33
TCGA-ZG-A9L4	censored	36.4
TCGA-EJ-5525	censored	36.63
TCGA-EJ-7791	censored	36.73
TCGA-XK-AAJR	censored	36.93
TCGA-XJ-A83F	censored	37.02
TCGA-V1-A9OX	censored	37.25

TCGA-HC-7081	censored	37.32
TCGA-EJ-7314	censored	37.78
TCGA-ZG-A9L2	censored	37.91
TCGA-G9-6378	censored	37.94
TCGA-G9-6370	censored	37.98
TCGA-EJ-8470	censored	38.07
TCGA-HC-7232	censored	38.14
TCGA-EJ-7782	censored	38.34
TCGA-EJ-5502	censored	38.47
TCGA-EJ-7785	censored	38.67
TCGA-HC-7079	censored	38.9
TCGA-V1-A9OF	censored	38.9
TCGA-G9-7510	censored	38.93
TCGA-V1-A8MM	censored	39.26
TCGA-G9-6364	censored	39.36
TCGA-G9-6369	censored	39.91
TCGA-XK-AAIW	censored	40.01
TCGA-J4-8200	censored	40.01
TCGA-HC-7213	censored	40.05
TCGA-HC-7211	censored	40.11
TCGA-G9-6367	censored	40.14
TCGA-G9-6371	censored	40.28
TCGA-J9-A8CN	censored	40.64
TCGA-QU-A6IM	censored	40.97
TCGA-XK-AAJP	censored	41.36
TCGA-VN-A88P	censored	41.39
TCGA-YL-A8SC	censored	41.43
TCGA-XK-AAJ3	censored	41.46
TCGA-G9-6329	censored	41.59
TCGA-2A-AAYO	censored	41.79
TCGA-EJ-5531	censored	41.82
TCGA-XJ-A83H	censored	42.05
TCGA-ZG-A9L1	censored	42.77
TCGA-VP-A87K	censored	42.9
TCGA-EJ-7794	censored	42.9
TCGA-KC-A4BV	censored	43.63
TCGA-KK-A7B0	censored	43.66
TCGA-CH-5762	censored	43.99
TCGA-KC-A4BR	censored	44.32
TCGA-HC-7233	censored	44.51
TCGA-V1-A9ZR	censored	44.71
TCGA-G9-6365	censored	44.78
TCGA-2A-AAYF	censored	44.81
TCGA-VP-AA1N	censored	44.84
TCGA-EJ-5501	censored	44.84
TCGA-VN-A88Q	censored	44.88
TCGA-YL-A8SF	censored	45.11
TCGA-2A-A8VT	censored	45.11
TCGA-G9-6363	censored	45.27
TCGA-2A-A8VX	censored	45.27
TCGA-EJ-5506	censored	45.37
TCGA-X4-A8KQ	censored	45.43
TCGA-EJ-5503	censored	45.7
TCGA-YL-A8SJ	censored	45.83

TCGA-EJ-7792	censored	45.86
TCGA-YL-A8SK	censored	46.25
TCGA-G9-6361	censored	46.48
TCGA-EJ-5499	censored	46.48
TCGA-EJ-5504	censored	46.88
TCGA-G9-6356	censored	47.11
TCGA-XK-AAJT	censored	47.11
TCGA-G9-6362	censored	47.4
TCGA-V1-A8MF	censored	47.6
TCGA-XJ-A9DI	censored	48
TCGA-XJ-A83G	censored	48
TCGA-EJ-5509	censored	48.09
TCGA-EJ-5511	censored	48.49
TCGA-EJ-5494	censored	48.52
TCGA-YL-A8HM	censored	48.69
TCGA-KK-A6E0	censored	48.72
TCGA-YL-A8HK	censored	48.98
TCGA-EJ-5542	censored	49.7
TCGA-G9-6348	censored	49.77
TCGA-V1-A9ZG	censored	49.84
TCGA-YL-A9WX	censored	49.87
TCGA-G9-6353	censored	50.66
TCGA-G9-6499	censored	50.69
TCGA-KK-A8IJ	censored	51.22
TCGA-YL-A8HL	censored	51.41
TCGA-XK-AAJU	censored	51.77
TCGA-G9-7509	censored	52.89
TCGA-YL-A8HJ	censored	52.92
TCGA-YL-A8SI	censored	53.02
TCGA-YL-A9WI	censored	53.32
TCGA-V1-A9ZI	censored	53.88
TCGA-VP-A87C	censored	55.52
TCGA-G9-6342	censored	55.72
TCGA-2A-A8VO	censored	55.88
TCGA-YL-A8SB	censored	56.47
TCGA-EJ-5498	censored	56.5
TCGA-G9-6496	censored	56.7
TCGA-EJ-5512	censored	56.93
TCGA-KK-A7AY	censored	57.62
TCGA-EJ-5495	censored	57.82
TCGA-KK-A6E8	censored	58.15
TCGA-G9-6494	censored	58.18
TCGA-VP-A87J	censored	58.28
TCGA-KK-A7AU	censored	58.31
TCGA-G9-6379	censored	58.41
TCGA-V1-A8MJ	censored	58.84
TCGA-KC-A4BN	censored	59.63
TCGA-EJ-5514	censored	60.09
TCGA-EJ-5530	censored	60.18
TCGA-EJ-5515	censored	60.18
TCGA-YL-A8S9	censored	60.51
TCGA-VP-A87D	censored	60.87
TCGA-V1-A8MU	censored	61.63
TCGA-EJ-5524	censored	61.83

TCGA-EJ-5510	censored	61.99
TCGA-EJ-5516	censored	62.06
TCGA-EJ-5517	censored	62.06
TCGA-YL-A8S8	censored	62.09
TCGA-EJ-5526	censored	62.71
TCGA-V1-A9OL	censored	62.84
TCGA-KK-A8IM	censored	62.91
TCGA-V1-A9O5	censored	63.47
TCGA-G9-6366	censored	63.96
TCGA-V1-A8WL	censored	63.99
TCGA-G9-6498	censored	64.13
TCGA-EJ-5519	censored	64.45
TCGA-EJ-5508	censored	64.55
TCGA-HC-7078	censored	65.21
TCGA-KK-A8I7	censored	65.77
TCGA-KK-A8IH	censored	66.13
TCGA-G9-6338	censored	66.62
TCGA-VP-A87E	censored	66.92
TCGA-G9-6351	censored	67.28
TCGA-KK-A8IC	censored	67.38
TCGA-KK-A8IK	censored	67.41
TCGA-KK-A6E3	censored	67.54
TCGA-G9-6354	censored	67.87
TCGA-G9-6336	censored	67.94
TCGA-KK-A6E5	censored	68.1
TCGA-EJ-5522	censored	68.3
TCGA-YL-A8SQ	censored	68.43
TCGA-G9-6347	censored	68.63
TCGA-KK-A8ID	censored	69.22
TCGA-HC-7077	censored	69.48
TCGA-EJ-5518	censored	69.58
TCGA-YL-A9WK	censored	70.17
TCGA-KK-A6E1	censored	72.21
TCGA-KK-A8I4	censored	74.21
TCGA-EJ-5521	censored	74.87
TCGA-YL-A8HO	censored	75.69
TCGA-VP-A875	censored	75.85
TCGA-EJ-8468	censored	77.1
TCGA-YL-A8SP	censored	77.73
TCGA-V1-A9OH	censored	78.94
TCGA-G9-6343	censored	80.49
TCGA-G9-6333	censored	80.98
TCGA-KK-A5A1	deceased	81.11
TCGA-V1-A9O7	censored	82.1
TCGA-KK-A8IG	censored	82.29
TCGA-HI-7170	censored	82.85
TCGA-KK-A59X	censored	83.28
TCGA-EJ-7218	censored	83.51
TCGA-G9-6339	censored	83.8
TCGA-EJ-7123	censored	84.49
TCGA-QU-A6IP	censored	86.07
TCGA-KK-A59Z	censored	86.33
TCGA-G9-6332	censored	87.29
TCGA-HI-7169	censored	88.17

TCGA-EJ-7115	censored	88.27
TCGA-VP-A87B	censored	89.42
TCGA-KK-A6E7	censored	90.67
TCGA-EJ-7125	censored	93.63
TCGA-V1-A9O9	censored	94.28
TCGA-KK-A8I5	censored	101.71
TCGA-VP-A878	censored	102.83
TCGA-VP-A876	censored	109.49
TCGA-KK-A59V	censored	113.01
TCGA-KK-A6E6	censored	113.24
TCGA-KK-A8II	deceased	113.9
TCGA-YL-A8SO	censored	114.29
TCGA-KK-A6E4	deceased	115.05
TCGA-VP-A872	censored	119.28
TCGA-QU-A6IO	censored	122.08
TCGA-QU-A6IN	censored	140.08
TCGA-KK-A6DY	censored	141.1
TCGA-KK-A6E2	censored	165.05
TCGA-YL-A9WJ	deceased	60.94
TCGA-ZG-A9LU	censored	19.02
TCGA-CH-5792	censored	2.99

Table S3

TCGA		mCRPC-SU2C/PFC Dream '		P value
PATIENT_ID	PCAT1	PATIENT_ID	PCAT1	8.7512E-05
TCGA-2A-A8VL-01	0.2259	TP_2061	1.21019	
TCGA-2A-A8VO-01	3.438038	SC_9091	5.87397	
TCGA-2A-A8VT-01	1.147885	SC_9086	0.440978	
TCGA-2A-A8VV-01	1.183039	MO_1339	0.045566	
TCGA-2A-A8VX-01	4.813374	MO_1337	3.86325	
TCGA-2A-A8W1-01	4.425281	MO_1336	1.93941	
TCGA-2A-A8W3-01	0.819681	SC_9031	0.093509	
TCGA-2A-AAYF-01	0.819126	SC_9081	0.963777	
TCGA-2A-AAYO-01	3.229443	SC_9080	0.506829	
TCGA-2A-AAYU-01	2.683579	MO_1316	2.83768	
TCGA-4L-AA1F-01	0.434795	TP_2054	4.45353	
TCGA-CH-5737-01	0.60554	SC_9073	0.246794	
TCGA-CH-5738-01	0.758352	SC_9072	24.2192	
TCGA-CH-5739-01	0.737263	SC_9071	1.98907	
TCGA-CH-5740-01	0.929802	SC_9068	3.61248	
TCGA-CH-5741-01	0.293311	MO_1277	2.15789	
TCGA-CH-5743-01	0.249736	MO_1202	2.7614	
TCGA-CH-5744-01	0.456151	SC_9063	4.2715	
TCGA-CH-5745-01	1.272028	SC_9062	10.3473	
TCGA-CH-5746-01	0.270696	SC_9061	1.39	
TCGA-CH-5748-01	3.222211	MO_1262	38.1222	
TCGA-CH-5750-01	7.668671	MO_1249	0.381055	
TCGA-CH-5751-01	20.81741	MO_1244	0.184884	
TCGA-CH-5752-01	5.521087	MO_1241	22.6079	
TCGA-CH-5753-01	0.66809	SC_9060	0.99169	
TCGA-CH-5754-01	0.607355	SC_9059	1.64928	
TCGA-CH-5761-01	0.020714	SC_9058	3.10646	
TCGA-CH-5762-01	2.769762	SC_9057	0.501842	
TCGA-CH-5763-01	0.334773	MO_1232	0.712733	
TCGA-CH-5764-01	1.327876	MO_1221	5.48685	
TCGA-CH-5765-01	2.492453	MO_1219	0.28582	
TCGA-CH-5766-01	0.75425	MO_1215	0.431676	
TCGA-CH-5767-01	3.298118	SC_9055	21.4861	
TCGA-CH-5768-01	2.169591	SC_9038	5.3697	
TCGA-CH-5769-01	1.391206	SC_9050	7.43448	
TCGA-CH-5771-01	0.809347	MO_1192	3.16119	
TCGA-CH-5772-01	1.616795	MO_1176	2.1508	
TCGA-CH-5788-01	2.673885	SC_9054	0.036412	
TCGA-CH-5789-01	0.247666	SC_9049	0.280111	
TCGA-CH-5790-01	2.404574	SC_9048	1.86135	
TCGA-CH-5791-01	2.226594	SC_9047	7.11884	
TCGA-CH-5792-01	0.348013	SC_9046	0.746945	
TCGA-CH-5794-01	1.203047	MO_1184	0.109045	
TCGA-EJ-5494-01	0.9588	MO_1179	0.519404	
TCGA-EJ-5495-01	0.351385	SC_9043	4.69112	
TCGA-EJ-5496-01	0.158437	SC_9018	1.2375	
TCGA-EJ-5497-01	0.522665	SC_9037	1.59004	
TCGA-EJ-5498-01	0.35057	SC_9036	7.80981	
TCGA-EJ-5499-01	3.526721	SC_9034	0.63529	
TCGA-EJ-5501-01	0.788922	SC_9032	1.47586	

TCGA-EJ-5502-01	1.379207	TP_2034	0.139439
TCGA-EJ-5503-01	0.18979	MO_1161	0.185819
TCGA-EJ-5504-01	5.689878	SC_9030	1.44004
TCGA-EJ-5505-01	0.537755	TP_2032	0.188815
TCGA-EJ-5506-01	0.688303	SC_9029	1.04093
TCGA-EJ-5507-01	0.99819	SC_9028	2.37386
TCGA-EJ-5508-01	0.688425	SC_9026	2.60865
TCGA-EJ-5509-01	1.540647	TP_2020	2.75545
TCGA-EJ-5510-01	0.453234	SC_9023	11.5104
TCGA-EJ-5511-01	2.341035	SC_9022	0.290762
TCGA-EJ-5512-01	1.951962	SC_9019	1.8125
TCGA-EJ-5514-01	0.462738	SC_9017	0.155759
TCGA-EJ-5515-01	1.626379	SC_9016	11.9931
TCGA-EJ-5516-01	0.965427	MO_1124	0.090441
TCGA-EJ-5517-01	1.72	MO_1128	1.83917
TCGA-EJ-5518-01	0.050217	MO_1118	0.167072
TCGA-EJ-5519-01	0.943414	MO_1114	6.95467
TCGA-EJ-5521-01	0.584406	SC_9012	0.10044
TCGA-EJ-5522-01	0.637636	SC_9007	3.25059
TCGA-EJ-5524-01	1.205028	TP_2009	17.5065
TCGA-EJ-5525-01	10.2138	TP_2010	0.039296
TCGA-EJ-5526-01	0.493661	SC_9010	1.44037
TCGA-EJ-5527-01	0.890433	SC_9009	2.11809
TCGA-EJ-5530-01	2.661496	SC_9008	4.56034
TCGA-EJ-5531-01	2.381007	TP_2001	1.93648
TCGA-EJ-5532-01	14.00157	MO_1095	0.867689
TCGA-EJ-5542-01	0.316974	SC_9001	0.238655
TCGA-EJ-7115-01	1.330367	MO_1094	0.138581
TCGA-EJ-7123-01	2.748333	MO_1084	4.86686
TCGA-EJ-7125-01	1.325691	MO_1071	3.54408
TCGA-EJ-7218-01	4.482796	MO_1054	2.19341
TCGA-EJ-7312-01	0.813488	MO_1040	0.228881
TCGA-EJ-7314-01	2.095781	MO_1020	0.370716
TCGA-EJ-7315-01	0.285195	MO_1014	0.658367
TCGA-EJ-7317-01	1.745849	MO_1013	5.04579
TCGA-EJ-7318-01	0.604974	TP_2060	21.4659
TCGA-EJ-7321-01	2.84146	SC_9083	8.68725
TCGA-EJ-7325-01	4.026656	TP_2064	8.70675
TCGA-EJ-7327-01	0.653191	SC_9097	1.52643
TCGA-EJ-7328-01	6.71817	SC_9093	1.80472
TCGA-EJ-7330-01	1.208039	SC_9092	5.15113
TCGA-EJ-7331-01	2.85816	SC_9099	0.221237
TCGA-EJ-7781-01	4.821855	SC_9094	5.02287
TCGA-EJ-7782-01	1.400833	6115251	0.428161
TCGA-EJ-7783-01	0.536917	6115247	1.10119
TCGA-EJ-7784-01	3.771792	6115242	0.334675
TCGA-EJ-7785-01	0.848931	6115219	0.886307
TCGA-EJ-7786-01	3.010544	6115122	3.15382
TCGA-EJ-7788-01	1.414731	6115114	8.08822
TCGA-EJ-7789-01	6.12031	1115202	0.372447
TCGA-EJ-7791-01	1.601267	6115117	3.03299
TCGA-EJ-7792-01	6.032563	6115115	1.63541
TCGA-EJ-7793-01	0.674543	1115161	18.7839
TCGA-EJ-7794-01	3.897588	1115183	7.27088

TCGA-EJ-7797-01	0.952695
TCGA-EJ-8468-01	7.524677
TCGA-EJ-8469-01	30.86606
TCGA-EJ-8470-01	2.78894
TCGA-EJ-8472-01	0.782125
TCGA-EJ-8474-01	2.980952
TCGA-EJ-A46B-01	1.026271
TCGA-EJ-A46D-01	0.792592
TCGA-EJ-A46E-01	0.706655
TCGA-EJ-A46F-01	0.648994
TCGA-EJ-A46G-01	3.514082
TCGA-EJ-A46H-01	0.219322
TCGA-EJ-A46I-01	1.512453
TCGA-EJ-A65B-01	0.027394
TCGA-EJ-A65D-01	2.381261
TCGA-EJ-A65E-01	1.288677
TCGA-EJ-A65F-01	1.301234
TCGA-EJ-A65G-01	2.214864
TCGA-EJ-A65J-01	1.058678
TCGA-EJ-A65M-01	2.130101
TCGA-EJ-A6RA-01	1.730725
TCGA-EJ-A6RC-01	1.963201
TCGA-EJ-A7NF-01	0.443892
TCGA-EJ-A7NG-01	0.774993
TCGA-EJ-A7NH-01	2.211282
TCGA-EJ-A7NJ-01	0.281174
TCGA-EJ-A7NK-01	0.477148
TCGA-EJ-A7NM-01	0.895572
TCGA-EJ-A7NN-01	0.156403
TCGA-EJ-A8FN-01	2.273643
TCGA-EJ-A8FO-01	0.461942
TCGA-EJ-A8FP-01	0.467003
TCGA-EJ-A8FS-01	9.525458
TCGA-EJ-A8FU-01	0.490439
TCGA-EJ-AB20-01	0.207666
TCGA-EJ-AB27-01	2.32578
TCGA-FC-7708-01	0.426391
TCGA-FC-7961-01	0.681051
TCGA-FC-A4JI-01	1.186088
TCGA-FC-A5OB-01	1.29271
TCGA-FC-A66V-01	0.090534
TCGA-FC-A6HD-01	5.146806
TCGA-FC-A8O0-01	0.113421
TCGA-G9-6329-01	0.348995
TCGA-G9-6332-01	0.653157
TCGA-G9-6333-01	1.455655
TCGA-G9-6336-01	0.175357
TCGA-G9-6338-01	2.242004
TCGA-G9-6339-01	0.790803
TCGA-G9-6342-01	0.691668
TCGA-G9-6343-01	1.534167
TCGA-G9-6347-01	2.653536
TCGA-G9-6348-01	1.071868
TCGA-G9-6351-01	0.47056

6115237	0.501447
6115233	2.79863
6115118	5.11024
6115123	0.096538
1115153	1.63
6115121	80.6767
1115156	3.68717
1115154	10.6659
6115227	1.23743
6115234	5.92868
6115224	18.868
1115244	1.47643
1115157	3.78433
6115250	0.191404

TCGA-G9-6353-01	0.367873
TCGA-G9-6354-01	0.670186
TCGA-G9-6356-01	1.193176
TCGA-G9-6361-01	1.148197
TCGA-G9-6362-01	0.320597
TCGA-G9-6363-01	0.53958
TCGA-G9-6364-01	3.699635
TCGA-G9-6365-01	0.675519
TCGA-G9-6366-01	0.082884
TCGA-G9-6367-01	0.277463
TCGA-G9-6369-01	1.641212
TCGA-G9-6370-01	0.113314
TCGA-G9-6371-01	1.92187
TCGA-G9-6373-01	2.75885
TCGA-G9-6377-01	5.027919
TCGA-G9-6378-01	1.01493
TCGA-G9-6379-01	0.592936
TCGA-G9-6384-01	1.03165
TCGA-G9-6385-01	1.162167
TCGA-G9-6494-01	2.365457
TCGA-G9-6496-01	1.309754
TCGA-G9-6498-01	1.646811
TCGA-G9-6499-01	1.165516
TCGA-G9-7509-01	0.439641
TCGA-G9-7510-01	2.327861
TCGA-G9-7519-01	2.812518
TCGA-G9-7521-01	0.447241
TCGA-G9-7522-01	0.248649
TCGA-G9-7523-01	3.728214
TCGA-G9-7525-01	1.120369
TCGA-G9-A9S0-01	0.165603
TCGA-G9-A9S4-01	19.13189
TCGA-G9-A9S7-01	2.526415
TCGA-H9-7775-01	0.579808
TCGA-H9-A6BX-01	0.157208
TCGA-H9-A6BY-01	1.929047
TCGA-HC-7075-01	12.12652
TCGA-HC-7077-01	6.064613
TCGA-HC-7078-01	1.291833
TCGA-HC-7079-01	0.150582
TCGA-HC-7080-01	1.084894
TCGA-HC-7081-01	0.243594
TCGA-HC-7209-01	0.441711
TCGA-HC-7210-01	0.284859
TCGA-HC-7211-01	5.652485
TCGA-HC-7212-01	2.904756
TCGA-HC-7213-01	0.35459
TCGA-HC-7230-01	2.429446
TCGA-HC-7231-01	3.541764
TCGA-HC-7232-01	1.621999
TCGA-HC-7233-01	2.160869
TCGA-HC-7736-01	0.182956
TCGA-HC-7737-01	1.618432
TCGA-HC-7738-01	0.757558

TCGA-HC-7740-01	2.759349
TCGA-HC-7740-01_r	0.591575
TCGA-HC-7742-01	0.482903
TCGA-HC-7744-01	0.597223
TCGA-HC-7745-01	0.572664
TCGA-HC-7747-01	0.564336
TCGA-HC-7748-01	1.211199
TCGA-HC-7749-01	1.547989
TCGA-HC-7750-01	2.193493
TCGA-HC-7752-01	6.525429
TCGA-HC-7817-01	0.979747
TCGA-HC-7818-01	2.781399
TCGA-HC-7819-01	1.288933
TCGA-HC-7820-01	7.589661
TCGA-HC-7821-01	0.816884
TCGA-HC-8213-01	2.095746
TCGA-HC-8216-01	4.330983
TCGA-HC-8256-01	10.85034
TCGA-HC-8257-01	0.690694
TCGA-HC-8258-01	1.303956
TCGA-HC-8258-01_r	1.793729
TCGA-HC-8259-01	2.413515
TCGA-HC-8260-01	0.429307
TCGA-HC-8261-01	12.30436
TCGA-HC-8262-01	3.261996
TCGA-HC-8264-01	0.509688
TCGA-HC-8265-01	1.995101
TCGA-HC-8265-01_r	3.330592
TCGA-HC-8266-01	0.689941
TCGA-HC-A48F-01	2.238057
TCGA-HC-A4ZV-01	2.053179
TCGA-HC-A631-01	0.695622
TCGA-HC-A632-01	0.757257
TCGA-HC-A6AL-01	1.052765
TCGA-HC-A6AN-01	0.589016
TCGA-HC-A6AO-01	0.718722
TCGA-HC-A6AP-01	0.477524
TCGA-HC-A6AQ-01	2.534396
TCGA-HC-A6AS-01	1.498316
TCGA-HC-A6HX-01	0.687057
TCGA-HC-A6HY-01	0.624755
TCGA-HC-A76W-01	0.487938
TCGA-HC-A76X-01	1.850083
TCGA-HC-A8CY-01	1.863198
TCGA-HC-A8D0-01	0.521952
TCGA-HC-A8D1-01	1.308608
TCGA-HC-A9TE-01	1.169128
TCGA-HC-A9TH-01	0.440378
TCGA-HI-7168-01	2.27721
TCGA-HI-7169-01	0.91252
TCGA-HI-7170-01	3.002924
TCGA-HI-7171-01	1.807219
TCGA-J4-8198-01	0.941413
TCGA-J4-8200-01	5.410665

TCGA-J4-A67K-01	7.72201
TCGA-J4-A67L-01	1.762813
TCGA-J4-A67M-01	3.164649
TCGA-J4-A67N-01	1.932776
TCGA-J4-A67O-01	0.406045
TCGA-J4-A67Q-01	1.927794
TCGA-J4-A67R-01	0.37001
TCGA-J4-A67S-01	0.754561
TCGA-J4-A67T-01	0.600101
TCGA-J4-A6G1-01	0.228775
TCGA-J4-A6G3-01	2.079902
TCGA-J4-A6M7-01	0.5045
TCGA-J4-A83I-01	0.676191
TCGA-J4-A83J-01	3.175044
TCGA-J4-A83K-01	1.690605
TCGA-J4-A83L-01	1.526996
TCGA-J4-A83M-01	3.08455
TCGA-J4-A83N-01	0.787916
TCGA-J4-AATV-01	0.651981
TCGA-J4-AATZ-01	0.591521
TCGA-J4-AAU2-01	1.739231
TCGA-J9-A52B-01	0.17096
TCGA-J9-A52C-01	0.138524
TCGA-J9-A52D-01	18.84462
TCGA-J9-A52E-01	1.733469
TCGA-J9-A8CK-01	4.571992
TCGA-J9-A8CL-01	10.95722
TCGA-J9-A8CM-01	1.603508
TCGA-J9-A8CN-01	0.996065
TCGA-J9-A8CP-01	1.231179
TCGA-KC-A4BL-01	14.11029
TCGA-KC-A4BN-01	0.888658
TCGA-KC-A4BR-01	0.692105
TCGA-KC-A4BV-01	0.591472
TCGA-KC-A7F3-01	2.211649
TCGA-KC-A7F5-01	1.262071
TCGA-KC-A7F6-01	3.991976
TCGA-KC-A7FA-01	2.993149
TCGA-KC-A7FD-01	0.59104
TCGA-KC-A7FE-01	0.910575
TCGA-KK-A59V-01	0.496122
TCGA-KK-A59X-01	0.65213
TCGA-KK-A59Y-01	1.293084
TCGA-KK-A59Z-01	3.201177
TCGA-KK-A5A1-01	5.493862
TCGA-KK-A6DY-01	1.442105
TCGA-KK-A6E0-01	6.094115
TCGA-KK-A6E1-01	2.453609
TCGA-KK-A6E2-01	1.076705
TCGA-KK-A6E3-01	0.169586
TCGA-KK-A6E4-01	0.749077
TCGA-KK-A6E5-01	2.683733
TCGA-KK-A6E6-01	0.504345
TCGA-KK-A6E7-01	0.056344

TCGA-KK-A6E8-01	6.236335
TCGA-KK-A7AP-01	0.817254
TCGA-KK-A7AQ-01	2.989547
TCGA-KK-A7AU-01	0.487075
TCGA-KK-A7AV-01	2.079817
TCGA-KK-A7AW-01	2.875377
TCGA-KK-A7AY-01	0.64773
TCGA-KK-A7AZ-01	5.023051
TCGA-KK-A7B0-01	2.289279
TCGA-KK-A7B1-01	2.571925
TCGA-KK-A7B2-01	0.696657
TCGA-KK-A7B3-01	1.061922
TCGA-KK-A7B4-01	1.536245
TCGA-KK-A8I4-01	0.568498
TCGA-KK-A8I5-01	3.612101
TCGA-KK-A8I6-01	2.52325
TCGA-KK-A8I7-01	0.162342
TCGA-KK-A8I8-01	7.726312
TCGA-KK-A8I9-01	2.314398
TCGA-KK-A8IA-01	2.76475
TCGA-KK-A8IB-01	2.091297
TCGA-KK-A8IC-01	0.623261
TCGA-KK-A8ID-01	1.600147
TCGA-KK-A8IF-01	21.56785
TCGA-KK-A8IG-01	1.705971
TCGA-KK-A8IH-01	4.144
TCGA-KK-A8II-01	3.195909
TCGA-KK-A8IJ-01	0.817136
TCGA-KK-A8IK-01	0.544731
TCGA-KK-A8IL-01	1.569842
TCGA-KK-A8IM-01	0.75545
TCGA-M7-A71Y-01	1.183132
TCGA-M7-A71Z-01	0.591753
TCGA-M7-A720-01	0.331853
TCGA-M7-A721-01	1.375234
TCGA-M7-A722-01	4.064497
TCGA-M7-A723-01	3.069803
TCGA-M7-A724-01	0.535324
TCGA-M7-A725-01	1.774975
TCGA-MG-AAMC-01	1.198604
TCGA-QU-A6IL-01	2.780775
TCGA-QU-A6IM-01	3.05866
TCGA-QU-A6IN-01	5.204128
TCGA-QU-A6IO-01	2.840604
TCGA-QU-A6IP-01	0.469669
TCGA-SU-A7E7-01	0.670741
TCGA-TK-A8OK-01	0.698971
TCGA-TP-A8TT-01	0.255056
TCGA-TP-A8TV-01	1.952823
TCGA-V1-A8MF-01	0.08841
TCGA-V1-A8MG-01	8.05594
TCGA-V1-A8MK-01	1.538121
TCGA-V1-A8ML-01	4.603684
TCGA-V1-A8MM-01	0.876218

TCGA-V1-A8MU-01	9.662003
TCGA-V1-A8WL-01	2.541721
TCGA-V1-A8WN-01	0.534031
TCGA-V1-A8WS-01	1.331702
TCGA-V1-A8WV-01	2.103699
TCGA-V1-A8WW-01	1.136432
TCGA-V1-A8X3-01	1.039178
TCGA-V1-A9O5-01	2.323111
TCGA-V1-A9O7-01	1.453511
TCGA-V1-A9O9-01	1.774777
TCGA-V1-A9OA-01	0.499674
TCGA-V1-A9OF-01	4.016846
TCGA-V1-A9OH-01	2.231264
TCGA-V1-A9OL-01	0.549933
TCGA-V1-A9OQ-01	1.043354
TCGA-V1-A9OT-01	0.940947
TCGA-V1-A9OX-01	1.846798
TCGA-V1-A9OY-01	0.673734
TCGA-V1-A9Z7-01	21.46371
TCGA-V1-A9Z8-01	0.980792
TCGA-V1-A9Z9-01	0.932957
TCGA-V1-A9ZG-01	0.921387
TCGA-V1-A9ZI-01	4.165652
TCGA-V1-A9ZK-01	2.3524
TCGA-V1-A9ZR-01	4.654348
TCGA-VN-A88I-01	0.951771
TCGA-VN-A88K-01	6.122936
TCGA-VN-A88L-01	0.973814
TCGA-VN-A88M-01	0.861526
TCGA-VN-A88N-01	4.098917
TCGA-VN-A88O-01	7.729487
TCGA-VN-A88P-01	2.918949
TCGA-VN-A88Q-01	1.686272
TCGA-VN-A88R-01	2.293703
TCGA-VN-A943-01	1.593312
TCGA-VP-A872-01	0.549116
TCGA-VP-A875-01	4.985627
TCGA-VP-A876-01	2.043724
TCGA-VP-A878-01	0.466523
TCGA-VP-A879-01	0.629979
TCGA-VP-A87B-01	1.968465
TCGA-VP-A87C-01	1.998884
TCGA-VP-A87D-01	0.858187
TCGA-VP-A87E-01	1.707093
TCGA-VP-A87H-01	6.531034
TCGA-VP-A87J-01	0.465558
TCGA-VP-A87K-01	4.771067
TCGA-VP-AA1N-01	0.611417
TCGA-WW-A8ZI-01	1.681322
TCGA-X4-A8KQ-01	0.478817
TCGA-X4-A8KS-01	2.657944
TCGA-XA-A8JR-01	3.026187
TCGA-XJ-A83F-01	0.949723
TCGA-XJ-A83G-01	5.056538

TCGA-XJ-A83H-01	5.409285
TCGA-XJ-A9DI-01	0.867413
TCGA-XJ-A9DK-01	1.79357
TCGA-XJ-A9DQ-01	1.22231
TCGA-XJ-A9DX-01	2.117885
TCGA-XK-AAIR-01	0.891242
TCGA-XK-AAIV-01	0.45374
TCGA-XK-AAIW-01	0.227641
TCGA-XK-AAJ3-01	5.030544
TCGA-XK-AAJA-01	4.96918
TCGA-XK-AAJP-01	1.748304
TCGA-XK-AAJR-01	2.790313
TCGA-XK-AAJT-01	0.683629
TCGA-XK-AAJU-01	0.459958
TCGA-XK-AAK1-01	2.307111
TCGA-XQ-A8TA-01	0.788951
TCGA-XQ-A8TB-01	7.397756
TCGA-Y6-A8TL-01	6.43074
TCGA-Y6-A9XI-01	0.380117
TCGA-YJ-A8SW-01	4.368036
TCGA-YL-A8HJ-01	4.440703
TCGA-YL-A8HK-01	0.613216
TCGA-YL-A8HL-01	0.409056
TCGA-YL-A8HM-01	3.209196
TCGA-YL-A8HO-01	1.444405
TCGA-YL-A8S8-01	1.332514
TCGA-YL-A8S9-01	2.477728
TCGA-YL-A8SA-01	0.413659
TCGA-YL-A8SB-01	0.273583
TCGA-YL-A8SC-01	1.405517
TCGA-YL-A8SH-01	0.813203
TCGA-YL-A8SI-01	1.713285
TCGA-YL-A8SJ-01	0.398814
TCGA-YL-A8SK-01	0.329015
TCGA-YL-A8SL-01	1.419563
TCGA-YL-A8SO-01	5.291191
TCGA-YL-A8SP-01	2.504651
TCGA-YL-A8SQ-01	1.593639
TCGA-YL-A8SR-01	1.322335
TCGA-YL-A9WH-01	4.103433
TCGA-YL-A9WI-01	3.691009
TCGA-YL-A9WJ-01	0.160914
TCGA-YL-A9WK-01	58.32646
TCGA-YL-A9WL-01	0.332283
TCGA-YL-A9WX-01	0.405123
TCGA-YL-A9WY-01	2.566413
TCGA-ZG-A8QW-01	0.187901
TCGA-ZG-A8QX-01	1.057361
TCGA-ZG-A8QY-01	3.290955
TCGA-ZG-A8QZ-01	1.823467
TCGA-ZG-A9KY-01	0.706383
TCGA-ZG-A9L0-01	1.656791
TCGA-ZG-A9L1-01	0.803927
TCGA-ZG-A9L2-01	0.292244

TCGA-ZG-A9L4-01	0.992402
TCGA-ZG-A9L5-01	2.629748
TCGA-ZG-A9L6-01	0.739985
TCGA-ZG-A9L9-01	0.663149
TCGA-ZG-A9LB-01	33.81048
TCGA-ZG-A9LM-01	1.455966
TCGA-ZG-A9LN-01	3.61671
TCGA-ZG-A9LS-01	0.081267
TCGA-ZG-A9LU-01	0.053504
TCGA-ZG-A9LY-01	0.763641
TCGA-ZG-A9LZ-01	2.157645
TCGA-ZG-A9M4-01	0.47253
TCGA-ZG-A9MC-01	1.098698
TCGA-ZG-A9N3-01	0.846635
TCGA-ZG-A9ND-01	1.496908
TCGA-ZG-A9NI-01	1.328973

Table S4

#Transcript biotype: protein_coding
#Condition pairs: KD_vs_C

Column A: Track_id, The database name in transcript level.
Column B: Gene_Name, The name of gene.
Column C: Trans_Name, The name of transcript.
Column D: Locus, The genomic coordinates of transcript.
Column E: Strand, The strand of transcript.
Column F: Trans_Type, The biological type of transcript.
Column G: log2(fold_change), if the comparison is test vs control, log2 of the fold change will be calculated by Test_FPKM - Control_FPKM.
Column H: Fold_Change, 2^(log2(fold_change)).
Column I: p_value, The p-value of the F-statistic for Transcript. The p-value will be setted as 1 if any group in the comparison has no repilicate.
Column J: q_value, The FDR adjusted p-value. The p-value will be setted as 1 if any group in the comparison has no repilicate.
Column K, L: Group FPKM, The average of log scaled FPKM: log2(FPKM + 1) of transcript in groups.
Column M ~ R: FPKM, The FPKM of transcript in samples.

Track_id	Gene_Name	Trans_Name	Locus	Strand	Trans_Type	log2(fold_change)	Fold_Change	p_value	q_value	KD_FPKM	C_FPKM	C1	C2	C3	KD1	KD2	KD3
ENST00000361445.8_2	MTOR	MTOR-001	chr1:11166592-11322564	-	protein_coding	-0.569732791	0.673741564	0.00068	0.1071826	3.600259573	4.1699924	16.355404	17.311937	17.353096	10.562183	10.821123	12.051481
ENST00000240100.6_1	DUSP4	DUSP4-001	chr8:29190581-29208185	-	protein_coding	-2.35121525	0.195980871	4.757E-05	0.0360168	3.828500063	6.1797153	81.296486	74.370331	60.412575	13.294047	13.052929	13.274405
ENST00000240101.2_1	DUSP4	DUSP4-002	chr8:29194056-29206322	-	protein_coding	-0.7714933	0.585810802	0.0014897	0.1552161	0.198508168	0.9700015	0.94123	0.747667	1.215455	0.162758	0.136544	0.143392
ENST00000356207.9_1	FGFR1	FGFR1-004	chr8:38270248-38326134	-	protein_coding	-0.535324675	0.690003376	0.0081892	0.294065	0.180385999	0.7157107	0.497108	0.57741	0.875657	0.128762	0.204045	0.07068
ENST00000490882.5_1	FLNB	FLNB-002	chr3:57994127-58156501	+	protein_coding	-1.889776208	0.269848915	0.0190723	0.3859254	3.647988386	5.5377646	83.663445	24.904943	44.711048	10.56033	12.31231	11.80083
ENST00000370986.8_1	GADD45A	GADD45A-001	chr1:68150744-68154021	+	protein_coding	-0.898881869	0.53630222	0.0006698	0.1067737	3.752283016	4.6511649	26.506882	22.190197	23.869934	12.99192	11.382675	13.123991
ENST00000215631.8_1	GADD45B	GADD45B-001	chr19:2476125-2478257	+	protein_coding	-0.842668344	0.557611282	0.0003532	0.0855005	1.703319625	2.545988	4.82985	4.362805	5.370989	2.304996	2.16298	2.30357
ENST00000397979.3_1	MAP2K7	MAP2K7-002	chr19:7968776-7979363	+	protein_coding	-1.3485428	0.392688485	0.0014637	0.1534734	2.422192881	3.7707357	10.660089	14.514956	13.055983	3.799618	5.347945	4.053803
ENST00000329492.5_1	MAPK8IP2	MAPK8IP2-001	chr22:51039114-51049979	+	protein_coding	-1.366576899	0.38781032	0.041283	0.4944923	0.58377974	1.9503566	2.221617	1.524163	6.098331	0.580806	0.625487	0.310218
ENST00000318065.9_1	NFATC1	NFATC1-003	chr18:77160322-77289323	+	protein_coding	-0.921699676	0.527886737	0.037681	0.4813289	0.305474704	1.2271744	1.021163	0.960311	2.238341	0	0.571173	0.201288
ENST00000290472.3_1	PLA2G4D	PLA2G4D-001	chr15:42359881-42386752	-	protein_coding	-1.21454454	0.430909097	0.0039442	0.2297569	1.086801924	2.3013465	3.680158	3.055605	5.309672	1.286122	1.192005	0.912225
ENST00000413366.7_1	PRKCA	PRKCA-002	chr17:64298944-64806861	+	protein_coding	-0.801473127	0.573763012	0.00933	0.3088016	1.862420378	2.6638935	6.548075	5.465824	4.215254	2.616463	2.344079	2.975325
ENST00000353379.11_1	CDK10	CDK10-001	chr16:89753076-89762772	+	protein_coding	-1.180632935	0.441157912	0.0294763	0.4414386	2.105436868	3.2860698	9.098814	10.325142	7.115265	5.711039	2.720839	2.191305
ENST00000485972.5_1	CDK5	CDK5-001	chr7:150750899-150755617	-	protein_coding	-0.529349519	0.692867063	0.0162969	0.3689388	3.752261723	4.2816112	17.068081	18.313114	20.082199	14.711855	10.971245	12.009578
ENST00000357227.8_1	CDK16	CDK16-001	chrX:47078087-47089396	+	protein_coding	-0.408213344	0.753556012	0.0455066	0.5106692	5.109837684	5.518051	39.351383	42.05304	54.390385	34.460934	32.224144	33.949364
ENST00000584632.5_1	CDK12	CDK12-010	chr17:37617764-37682416	+	protein_coding	-0.362906242	0.777596569	0.0133509	0.3404724	1.314671766	1.677578	2.300253	1.944857	2.368158	1.646503	1.534692	1.294401
ENST00000341911.9_1	MYB	MYB-002	chr6:135502453-135540305	+	protein_coding	-0.420323102	0.747257252	0.0177371	0.3791403	0.163711812	0.5840349	0.444471	0.46326	0.593697	0.283301	0.058888	0.034351
ENST00000348956.6_1	CKB	CKB-001	chr14:103985996-103989448	-	protein_coding	-0.679986231	0.624171231	0.0051041	0.2517039	8.216612997	8.8965992	485.32788	499.92416	443.35342	293.93576	340.08286	260.6698
ENST00000557530.1_1	CKB	CKB-006	chr14:103988164-103989170	-	protein_coding	-1.45983159	0.363535563	0.0311133	0.4503103	0.734565216	2.1943968	6.882202	1.7828	3.371276	0.878892	0.453235	0.6871
ENST00000257566.7_2	TBX3	TBX3-002	chr12:115108060-115121395	-	protein_coding	-0.466944892	0.723495078	0.0001776	0.0650541	5.191025681	5.6579706	49.669277	47.832146	51.024277	36.575272	35.437336	34.605293
ENST00000263388.6_1	NOTCH3	NOTCH3-001	chr19:15269849-15311792	-	protein_coding	-0.949833493	0.517692207	0.0328534	0.4601417	2.898007577	3.8478411	11.812731	9.881369	20.410238	6.35481	6.175549	6.847567
ENST00000254958.9_1	JAG1	JAG1-002	chr20:10618332-10654755	-	protein_coding	-0.571512416	0.672910988	1.554E-05	0.0213921	3.655228668	4.2267411	18.040083	17.993269	17.149263	11.636132	11.515074	11.64599
ENST00000538183.6_1	SOD2	SOD2-001	chr6:160090089-160114360	-	protein_coding	-0.377212944	0.769923524	0.0368238	0.4772516	1.864145494	2.2413584	4.002987	4.014286	3.214149	3.121033	2.456919	2.386849
ENST00000286234.5_1	DEPTOR	DEPTOR-001	chr8:120885957-121063152	+	protein_coding	-0.971119347	0.510110129	0.0040297	0.2300711	2.565014252	3.5361336	9.577917	9.61446	12.904289	4.86691	4.282899	5.685855
ENST00000297494.7_1	NOS3	NOS3-001	chr7:150688083-150711676	+	protein_coding	-1.956155322	0.257714332	0.0004478	0.0946022	1.320099009	3.2762543	8.029095	7.314021	11.114267	1.634945	1.225933	1.6539
ENST00000520810.5_1	IKBKB	IKBKB-002	chr8:42128820-42189126	+	protein_coding	-1.273805748	0.413567365	0.0164201	0.3695439	1.24347635	2.5172821	6.104465	5.807282	2.879959	1.605194	1.659724	0.915554
ENST00000312629.9_1	RPS6KB2	RPS6KB2-003	chr11:67195972-67202879	+	protein_coding	-0.587469942	0.665508989	0.0482577	0.5155651	4.411530541	4.9990005	26.911291	27.661619	39.875835	19.586031	23.626781	18.012043
ENST00000520810.5_1	IKBKB	IKBKB-002	chr8:42128820-42189126	+	protein_coding	-1.273805748	0.413567365	0.0164201	0.3695439	1.24347635	2.5172821	6.104465	5.807282	2.879959	1.605194	1.659724	0.915554
ENST00000566846.5_1	ALDOA	ALDOA-030	chr16:30077179-30080213	+	protein_coding	-3.426760198	0.092991316	0.0406911	0.4919904	1.633184108	5.0599443	63.533619	67.08242	7.448197	0.898819	5.90927	1.275079
ENST00000395240.7_1	ALDOA	ALDOA-011	chr16:30077098-30081701	+	protein_coding	-0.485046574	0.714474004	0.0003419	0.0838816	6.673936406	7.158983	146.70641	145.56532	133.82651	102.06326	100.50324	100.76099
ENST00000569798.5_1	ALDOA	ALDOA-010	chr16:30077093-30081673	+	protein_coding	-0.227268598	0.854250683	0.022408	0.4027544	7.310617596	7.5378862	194.40109	171.35523	189.56279	163.48621	157.83392	152.13442
ENST00000358707.7_1	CAMK2B	CAMK2B-004	chr7:44258945-44365224	-	protein_coding	-1.549637359	0.341595918	0.0074624	0.2866558	1.333858749	2.8834961	4.974217	5.083773	10.056113	1.35683	1.335494	1.909963
ENST00000244741.9_1	CDKN1A	CDKN1A-001	chr6:36646435-36655116	+	protein_coding	-1.284378626	0.410547591	5.557E-06	0.0113405	5.1245859	6.4089645	86.441528	80.022766	85.605782	34.464882	32.942734	34.270927
ENST00000216117.8_1	HMOX1	HMOX1-001	chr22:35776828-35790207	+	protein_coding	-0.922926961	0.52743786	0.0207555	0.3951554	2.994375061	3.917302	18.163816	15.117182	10.166193	6.898031	6.13136	7.984604
ENST00000297494.7_1	NOS3	NOS3-001	chr7:150688083-150711676	+	protein_coding	-1.956155322	0.257714332	0.0004478	0.0946022	1.320099009	3.2762543	8.029095	7.314021	11.114267	1.634945	1.225933	1.6539
ENST00000413366.7_1	PRKCA	PRKCA-002	chr17:64298944-64806861	+	protein_coding	-0.801473127	0.573763012	0.00933	0.3088016	1.862420378	2.6638935	6.548075	5.465824	4.215254	2.616463	2.344079	2.975325
ENST00000425836.6_1	VEGFA	VEGFA-003	chr6:43738444-43752686	+	protein_coding	-0.502234048	0.706012657	0.049336	0.5198682	0.184311912	0.686546	0.475186	0.378111	1.050604	0.182932	0.093502	0.134151

AKT postively regulated gene

regulated gene

ENST00000297494.7_1	NOS3	NOS3-001	chr7:150688083-150711676	+	protein_coding	-1.956155322	0.257714332	0.0004478	0.0946022	1.320099009	3.2762543	8.029095	7.314021	11.114267	1.634945	1.225933	1.6539	NFKB postively
ENST00000252506.10_1	GADD45G	GADD45G-001	chr9:92219928-92221470	+	protein_coding	-0.957711426	0.514873019	0.0432347	0.5008314	3.424736101	4.3824475	13.74578	18.281502	30.910666	9.306825	9.827275	10.096878	
ENST00000371621.4_1	PTPN1	PTPN1-001	chr20:49126891-49201778	+	protein_coding	-0.180588745	0.88234285	0.025434	0.4228103	3.850657087	4.0312458	14.797493	15.80933	15.460354	13.255773	12.76663	14.299296	
ENST00000439267.1_1	BCL2L1	BCL2L1-005	chr20:30309479-30311792	-	protein_coding	-0.428634066	0.742964886	0.0237803	0.4118148	1.732548664	2.1611827	4.09938	3.095584	3.28459	2.313226	2.631206	2.05032	
ENST00000318065.9_1	NFATC1	NFATC1-003	chr18:77160322-77289323	+	protein_coding	-0.921699676	0.527886737	0.037681	0.4813289	0.305474704	1.2271744	1.021163	0.960311	2.238341	0	0.571173	0.201288	
ENST00000397979.3_1	MAP2K7	MAP2K7-002	chr19:7968776-7979363	+	protein_coding	-1.3485428	0.392688485	0.0014637	0.1534734	2.422192881	3.7707357	10.660089	14.514956	13.055983	3.799618	5.347945	4.053803	
ENST00000254958.9_1	JAG1	JAG1-002	chr20:10618332-10654755	-	protein_coding	-0.571512416	0.672910988	1.554E-05	0.0213921	3.655228668	4.2267411	18.040083	17.993269	17.149263	11.636132	11.515074	11.64599	
ENST00000249075.3_1	LIF	LIF-001	chr22:30636436-30642840	-	protein_coding	-0.583132845	0.667512683	0.0158793	0.3647047	1.459706332	2.0428392	3.057728	2.500936	3.924935	1.764603	1.833037	1.656814	
ENST00000440375.1_1	PDGFB	PDGFB-004	chr22:39626189-39637135	-	protein_coding	-0.764989595	0.588457613	0.0057315	0.2613922	1.348727439	2.113717	3.952671	2.752419	3.362404	1.280253	1.756371	1.628461	

Table S5

LncRNAs predicted by catRAPID omics module to interact with FKBP51 protein

Transcript ID	Z-score	Discriminative Power	Interaction Strength
ENST00000444330	2.28	99	100
ENST00000523265	2.25	99	100
ENST00000320165	3.06	100	100
ENST00000332587	2.45	99	100
ENST00000399492	2.47	99	100
ENST00000412321	3.01	100	100
ENST00000414722	3.57	100	100
ENST00000415106	2.36	99	100
ENST00000416366	2.35	99	100
ENST00000417483	2.72	99	100
ENST00000418699	2.52	99	100
ENST00000418821	2.5	99	100
ENST00000423499	2.7	99	100
ENST00000426495	2.32	99	100
ENST00000428699	3.06	100	100
ENST00000429798	3	100	100
ENST00000432330	4.02	100	100
ENST00000432361	2.49	99	100
ENST00000432473	2.59	99	100
ENST00000433079	3.04	100	100
ENST00000433465	3.31	100	100
ENST00000437514	2.4	99	100
ENST00000438753	2.61	99	100
ENST00000441722	2.38	99	100
ENST00000445785	2.91	100	100
ENST00000446213	2.24	99	100
ENST00000447260	2.99	100	100
ENST00000448344	2.42	99	100
ENST00000448579	2.24	99	100
ENST00000448942	2.56	99	100
ENST00000449579	2.42	99	100
ENST00000451101	2.76	99	100
ENST00000451744	3.09	100	100
ENST00000453309	2.44	99	100
ENST00000453698	2.62	99	100
ENST00000455253	2.62	99	100
ENST00000456327	2.72	99	100
ENST00000457147	2.65	99	100
ENST00000458351	3.24	100	100
ENST00000471299	3.44	100	100
ENST00000473550	2.26	99	100
ENST00000495576	2.43	99	100
ENST00000500324	2.25	99	100

ENST00000500496	3.28	100	100
ENST00000503066	2.49	99	100
ENST00000504349	2.47	99	100
ENST00000506086	2.3	99	100
ENST00000507296	3.73	100	100
ENST00000508732	4.48	100	100
ENST00000509015	3.64	100	100
ENST00000509295	2.56	99	100
ENST00000509788	2.44	99	100
ENST00000512624	3.45	100	100
ENST00000513955	2.28	99	100
ENST00000515428	2.48	99	100
ENST00000517898	2.94	100	100
ENST00000539315	3.37	100	100
ENST00000541359	2.23	99	100
ENST00000542980	4.64	100	100
ENST00000546612	4.25	100	100
ENST00000547175	2.69	99	100
ENST00000548268	3.14	100	100
ENST00000549796	4.25	100	100
ENST00000549905	2.72	100	100
ENST00000555332	2.77	100	100
ENST00000556899	2.46	99	100
ENST00000558010	2.6	99	100
ENST00000558945	2.35	99	100
ENST00000560187	3.94	100	100
ENST00000561215	2.31	99	100
ENST00000561978	2.53	99	100
ENST00000563046	2.53	99	100
ENST00000564199	2.22	99	100
ENST00000565797	2.25	99	100
ENST00000565798	2.3	99	100
ENST00000565841	4.23	100	100
ENST00000566297	2.5	99	100
ENST00000566900	3.16	100	100
ENST00000566922	2.23	99	100
ENST00000568332	2.53	99	100
ENST00000569694	2.85	100	100
ENST00000569865	3.31	100	100
ENST00000569908	2.48	99	100
ENST00000572964	2.96	100	100
ENST00000574628	2.3	99	100
ENST00000574741	2.19	99	100
ENST00000577806	3.24	100	100
ENST00000578206	2.8	100	100
ENST00000578977	2.32	99	100
ENST00000579492	3.52	100	100

ENST00000581174	3.04	100	100
ENST00000583253	2.26	99	100

Fold Change cut-off: **2.0**
# P-value cut-off: **0.01**
Condition pairs: **LNCaP-AI_vs_LNCaP**

Column A: ProbeName it represents probe name.
Column B: P-value, P-value calculated from unpaired t-test.
Column C: FDR, FDR is calculated from Benjamini Hochberg FDR.
Column D: Fold Change, the absolute ratio (no log scale) of normalized intensities between two conditions.
Column E: Regulation, it depicts which group has greater or lower intensity values wrt other group.
Column F ~ I: Annotations, include type, seqname, GeneSymbol, source

Note:
Column I: source, the source of LncRNA is collected from. GENCODE: the GENCODE project (http://www.gencodegenes.org/);

LNCaP-AI_vs_LNCaP 2.0 fold up regulated LncRNAs								
	P-value and FDR		Fold change and Regulation		Annotations			
ProbeName	P-value	FDR	Fold Change	Regulation	type	seqname	GeneSymbol	source
ASHGA5P034321	0.000667743	0.02272151	2.2742634	up	noncoding	ENST00000270443	ZNF702P	GENCODE
ASHGA5P028656	0.001230308	0.030074685	2.3238652	up	noncoding	ENST00000366259	LINC00449	GENCODE
ASHGA5P034837	6.47639E-07	0.001908013	22.7643969	up	noncoding	ENST00000366424	AC144450.2	GENCODE
ASHGA5P028402	0.003074422	0.04581914	3.3807578	up	noncoding	ENST00000376617	RP13-895J2.2	GENCODE
ASHGA5P013924	0.002791179	0.043560422	2.927504	up	noncoding	ENST00000397864	RP11-289F5.1	GENCODE
ASHGA5P014589	0.000674923	0.022743745	3.5221351	up	noncoding	ENST00000411615	RP11-356I2.1	GENCODE
ASHGA5P046686	0.000399816	0.017769355	8.9874628	up	noncoding	ENST00000412163	RP1-171K16.5	GENCODE
ASHGA5P014664	9.77067E-05	0.010379551	10.6047196	up	noncoding	ENST00000412276	AC007319.1	GENCODE
ASHGA5P014767	0.000107294	0.010443757	5.0473915	up	noncoding	ENST00000413202	LINC00152	GENCODE
ASHGA5P014769	0.000106036	0.010443757	3.7971094	up	noncoding	ENST00000413227	AC016995.3	GENCODE
ASHGA5P014825	0.001675549	0.033630672	2.786569	up	noncoding	ENST00000413745	RP13-143G15.3	GENCODE
ASHGA5P027985	0.006286419	0.065191665	2.2163787	up	noncoding	ENST00000415166	RP11-195M16.1	GENCODE
ASHGA5P015053	0.002303852	0.039484643	2.1143533	up	noncoding	ENST00000416068	PCBP1-AS1	GENCODE
ASHGA5P051225	0.00422435	0.052993068	2.009499	up	noncoding	ENST00000416405	LINC00281	GENCODE
ASHGA5P052713	4.20141E-05	0.007786911	220.0212666	up	noncoding	ENST00000416948	RP1-207H1.3	GENCODE
ASHGA5P037303	0.001459512	0.031508822	3.1815335	up	noncoding	ENST00000417138	DSCR4-IT1	GENCODE
ASHGA5P015168	0.002327141	0.039709543	4.6270202	up	noncoding	ENST00000417482	GRM7-AS3	GENCODE
ASHGA5P045128	0.002694249	0.042931807	3.8962013	up	noncoding	ENST00000417638	RP11-3L8.3	GENCODE
ASHGA5P046857	0.000128561	0.011376413	3.1352896	up	noncoding	ENST00000418196	RP11-292F22.5	GENCODE
ASHGA5P038998	0.000577207	0.02090606	21.989371	up	noncoding	ENST00000418242	RP11-148B18.3	GENCODE
ASHGA5P015244	0.00058679	0.02090606	2.4961175	up	noncoding	ENST00000418368	GUSBP4	GENCODE
ASHGA5P015250	6.25598E-06	0.00338644	12.5491885	up	noncoding	ENST00000418399	RP1-207H1.3	GENCODE
ASHGA5P028640	0.000597184	0.021097081	2.8196726	up	noncoding	ENST00000418607	BX004987.7	GENCODE
ASHGA5P034835	0.002534324	0.041486904	2.2854513	up	noncoding	ENST00000419028	AC141930.2	GENCODE
ASHGA5P038481	0.009391628	0.080127788	3.5858331	up	noncoding	ENST00000419450	RP5-952N6.1	GENCODE
ASHGA5P015385	4.64365E-05	0.007797264	3.1514707	up	noncoding	ENST00000419745	RP11-573D15.2	GENCODE
ASHGA5P048551	3.65625E-05	0.006961667	6.4520167	up	noncoding	ENST00000420153	AL928742.12	GENCODE
ASHGA5P037710	0.000365443	0.017237336	2.2644463	up	noncoding	ENST00000420537	RP1-85F18.5	GENCODE
ASHGA5P037763	0.000112656	0.010783994	11.0043956	up	noncoding	ENST00000420902	RP1-29C18.8	GENCODE
ASHGA5P015528	0.002986368	0.045021216	2.1354538	up	noncoding	ENST00000421071	AC068057.2	GENCODE
ASHGA5P050503	0.001045362	0.027678268	2.2279479	up	noncoding	ENST00000421255	PCBP1-AS1	GENCODE
ASHGA5P035165	0.000204861	0.01327973	3.3852921	up	noncoding	ENST00000421534	AC021188.4	GENCODE
ASHGA5P015646	1.82884E-05	0.005129549	13.3830772	up	noncoding	ENST00000422194	RP11-402P6.9	GENCODE
ASHGA5P028849	0.000598207	0.021097081	2.8854668	up	noncoding	ENST00000422527	RP11-168G22.3	GENCODE
ASHGA5P015751	0.001383282	0.031176625	3.7189353	up	noncoding	ENST00000423175	RP11-443B7.1	GENCODE
ASHGA5P039161	0.000339322	0.016746915	2.7078758	up	noncoding	ENST00000423466	RP11-114M1.1	GENCODE
ASHGA5P042949	0.00670556	0.067504568	2.9040244	up	noncoding	ENST00000423689	AC003090.1	GENCODE
ASHGA5P037820	0.003662651	0.049658142	2.2174421	up	noncoding	ENST00000424410	KB-1183D5.11	GENCODE
ASHGA5P037966	0.004552563	0.054813239	2.5369429	up	noncoding	ENST00000424761	RP4-633O19__A.1	GENCODE
ASHGA5P032753	0.001280981	0.030340524	8.3618033	up	noncoding	ENST00000425277	AC099684.1	GENCODE

ASHGA5P053209	9.74612E-06	0.003948764	2.9570418	up	noncoding	ENST00000425474	AZGP1P1	GENCODE
ASHGA5P035222	0.000456875	0.01904229	2.0824289	up	noncoding	ENST00000425576	AC011753.5	GENCODE
ASHGA5P016234	6.91459E-05	0.009485505	5.8400804	up	noncoding	ENST00000428329	AC007319.1	GENCODE
ASHGA5P041236	0.000652128	0.022409029	4.3444534	up	noncoding	ENST00000429352	RP11-107M16.2	GENCODE
ASHGA5P038751	0.003758784	0.050218975	2.9985601	up	noncoding	ENST00000431031	SPATA1	GENCODE
ASHGA5P016544	0.005172679	0.058499086	2.5707611	up	noncoding	ENST00000431488	LINC00281	GENCODE
ASHGA5P016557	0.000331625	0.016507355	4.0266647	up	noncoding	ENST00000431656	LINC00459	GENCODE
ASHGA5P035241	0.000331277	0.016507355	2.7787976	up	noncoding	ENST00000432268	AC017002.2	GENCODE
ASHGA5P016690	8.67178E-05	0.010071981	2.1588814	up	noncoding	ENST00000433005	AC003075.4	GENCODE
ASHGA5P016713	0.000566748	0.020743466	4.3891651	up	noncoding	ENST00000433296	AC007970.1	GENCODE
ASHGA5P046229	9.64631E-07	0.001908013	6.0610271	up	noncoding	ENST00000433410	RP11-402P6.9	GENCODE
ASHGA5P045335	0.002638877	0.042626293	2.4482031	up	noncoding	ENST00000433644	RP11-435O5.2	GENCODE
ASHGA5P033094	0.00044028	0.018753394	2.724824	up	noncoding	ENST00000433734	RP5-1065P14.2	GENCODE
ASHGA5P041993	0.001505705	0.032007146	3.6060396	up	noncoding	ENST00000434296	RP1-249H1.4	GENCODE
ASHGA5P048045	0.00671809	0.067524841	2.2087123	up	noncoding	ENST00000434346	RP11-184I16.2	GENCODE
ASHGA5P016803	0.000687956	0.022894499	2.8241098	up	noncoding	ENST00000434426	RP11-73B2.6	GENCODE
ASHGA5P035728	0.000817394	0.024691548	9.0474191	up	noncoding	ENST00000434509	AC092635.1	GENCODE
ASHGA5P037483	0.000355621	0.017162406	2.5041234	up	noncoding	ENST00000434589	AP001043.1	GENCODE
ASHGA5P016892	0.000296787	0.015726086	3.0850816	up	noncoding	ENST00000435434	RP11-127L20.6	GENCODE
ASHGA5P053156	0.006924671	0.068618554	2.1387304	up	noncoding	ENST00000435749	MAGI2-AS3	GENCODE
ASHGA5P053326	0.000558002	0.020689436	2.6107159	up	noncoding	ENST00000435996	AC009784.3	GENCODE
ASHGA5P043863	0.000207977	0.013370401	2.3408908	up	noncoding	ENST00000436097	AC002465.2	GENCODE
ASHGA5P037391	0.000101179	0.010443757	8.2937221	up	noncoding	ENST00000436429	AF127577.11	GENCODE
ASHGA5P017063	0.000878067	0.025327294	6.0938448	up	noncoding	ENST00000437232	RP11-124N14.4	GENCODE
ASHGA5P047208	0.000523116	0.020297836	3.1944186	up	noncoding	ENST00000437289	RP11-526P5.2	GENCODE
ASHGA5P015087	0.003641119	0.049443163	2.4447448	up	noncoding	ENST00000437456	PCBP1-AS1	GENCODE
ASHGA5P017086	0.000172361	0.012519564	4.9239724	up	noncoding	ENST00000437561	LINC00152	GENCODE
ASHGA5P045185	0.006348917	0.065477294	2.60074	up	noncoding	ENST00000437601	RP4-781K5.8	GENCODE
ASHGA5P017091	1.50248E-05	0.004560378	2.2526482	up	noncoding	ENST00000437608	AC093838.7	GENCODE
ASHGA5P034966	0.004148888	0.052874931	2.6174258	up	noncoding	ENST00000437680	AC020594.5	GENCODE
ASHGA5P045658	2.86536E-05	0.00636351	6.3757453	up	noncoding	ENST00000437830	XX-CR54.1	GENCODE
ASHGA5P036417	0.000256878	0.014691304	2.179819	up	noncoding	ENST00000437897	AC072062.3	GENCODE
ASHGA5P017135	0.000371025	0.017237336	5.8604922	up	noncoding	ENST00000438072	RP11-111F5.3	GENCODE
ASHGA5P042750	0.002117903	0.037660452	2.4610142	up	noncoding	ENST00000438217	XX-C2158C12.2	GENCODE
ASHGA5P017195	0.00069836	0.022956267	2.0033074	up	noncoding	ENST00000438770	AC012309.5	GENCODE
ASHGA5P037860	0.000183246	0.01271916	2.1569561	up	noncoding	ENST00000438893	AP000356.2	GENCODE
ASHGA5P046230	8.53381E-05	0.010071981	2.8631376	up	noncoding	ENST00000439926	RP11-402P6.11	GENCODE
ASHGA5P046502	0.001438422	0.031372189	2.6241529	up	noncoding	ENST00000440955	RP3-326I13.1	GENCODE
ASHGA5P035240	9.90549E-05	0.010395995	2.8410746	up	noncoding	ENST00000442293	AC068491.1	GENCODE
ASHGA5P017564	0.002827729	0.043751948	2.223549	up	noncoding	ENST00000443162	AC004854.4	GENCODE
ASHGA5P017636	0.001950153	0.036055957	2.843185	up	noncoding	ENST00000444032	AC002127.4	GENCODE
ASHGA5P042612	0.006035133	0.063492811	2.0100046	up	noncoding	ENST00000444229	RP11-527F13.1	GENCODE
ASHGA5P035471	0.003139393	0.046233731	4.3155054	up	noncoding	ENST00000444562	AC064871.3	GENCODE
ASHGA5P017740	4.7983E-05	0.007797264	19.0520707	up	noncoding	ENST00000445253	TTTY22	GENCODE
ASHGA5P034589	0.000579495	0.02090606	2.2741856	up	noncoding	ENST00000446262	AC092295.4	GENCODE
ASHGA5P027788	0.006730355	0.067582846	3.3462188	up	noncoding	ENST00000446693	RP11-24P14.1	GENCODE
ASHGA5P037380	0.002627962	0.042590091	2.4685565	up	noncoding	ENST00000448463	AP001347.6	GENCODE
ASHGA5P045246	7.12957E-06	0.00338644	3.9817405	up	noncoding	ENST00000448491	RP11-111F5.3	GENCODE
ASHGA5P041871	0.002677879	0.042905685	3.6904576	up	noncoding	ENST00000448991	RP1-214M20.2	GENCODE
ASHGA5P018122	0.001179844	0.029621209	3.0023601	up	noncoding	ENST00000449678	AC074289.1	GENCODE
ASHGA5P018176	4.79584E-05	0.007797264	3.7662951	up	noncoding	ENST00000450270	RP11-307B23.1	GENCODE
ASHGA5P045633	0.000216066	0.013664057	2.7466479	up	noncoding	ENST00000450445	RP11-62F24.2	GENCODE
ASHGA5P031650	0.000969899	0.026736674	2.6842045	up	noncoding	ENST00000450909	CTD-2574D22.2	GENCODE
ASHGA5P034716	0.009185201	0.079456094	2.5121893	up	noncoding	ENST00000451217	RP1-150O5.3	GENCODE
ASHGA5P050106	0.008728233	0.077464734	2.0890323	up	noncoding	ENST00000451691	CTD-2666L21.1	GENCODE
ASHGA5P018331	0.000847478	0.024940488	4.802226	up	noncoding	ENST00000452184	RP11-262H14.1	GENCODE
ASHGA5P018335	0.00148824	0.031895453	2.0725985	up	noncoding	ENST00000452249	AC003075.4	GENCODE
ASHGA5P030568	0.00319394	0.046481988	2.7636468	up	noncoding	ENST00000452467	AC025918.2	GENCODE
ASHGA5P036301	2.64491E-05	0.006259271	34.3882708	up	noncoding	ENST00000453517	AC007319.1	GENCODE
ASHGA5P018445	0.003689573	0.04983005	4.0658305	up	noncoding	ENST00000453639	RP11-598C10.2	GENCODE
ASHGA5P018499	0.00101342	0.027330972	4.5672944	up	noncoding	ENST00000454317	RP1-54A3.1	GENCODE
ASHGA5P036387	0.002918788	0.044471534	5.8265212	up	noncoding	ENST00000454444	AC007879.4	GENCODE
ASHGA5P018542	1.52031E-05	0.004560378	5.8207864	up	noncoding	ENST00000454780	RP11-538C21.1	GENCODE
ASHGA5P018571	0.000173488	0.012519564	4.818876	up	noncoding	ENST00000455131	LINC00152	GENCODE
ASHGA5P051422	0.005931827	0.063014806	2.31173	up	noncoding	ENST00000456253	AC144521.1	GENCODE
ASHGA5P018688	0.000423777	0.018456226	3.1452441	up	noncoding	ENST00000456532	RP5-1158E12.3	GENCODE
ASHGA5P016112	6.86794E-05	0.009485505	5.5321132	up	noncoding	ENST00000456541	TSPY5P	GENCODE
ASHGA5P045311	0.000864869	0.025117085	2.7026156	up	noncoding	ENST00000456944	RP11-82L18.2	GENCODE
ASHGA5P054217	0.001827921	0.035010194	2.1253004	up	noncoding	ENST00000457658	TTTY15	GENCODE
ASHGA5P018794	0.000246671	0.014421146	11.4081393	up	noncoding	ENST00000457716	RP11-402P6.7	GENCODE
ASHGA5P055674	0.008550487	0.076826498	2.0241205	up	noncoding	ENST00000457856	RP11-108M9.3	GENCODE
ASHGA5P054306	0.00335342	0.047167445	2.6803675	up	noncoding	ENST00000458044	RP11-443B7.1	GENCODE
ASHGA5P038523	0.006219805	0.064925974	3.950646	up	noncoding	ENST00000473352	RP11-392A22.2	GENCODE
ASHGA5P044289	0.000472357	0.019395401	2.9877114	up	noncoding	ENST00000476425	RP11-163N6.2	GENCODE
ASHGA5P019447	0.000713627	0.023195544	2.3775066	up	noncoding	ENST00000485041	TUBA4B	GENCODE
ASHGA5P019505	1.27694E-05	0.004560378	7.0824989	up	noncoding	ENST00000488584	RP11-392A22.2	GENCODE
ASHGA5P039080	0.003427915	0.047698091	2.1332321	up	noncoding	ENST00000489011	RP11-206M11.7	GENCODE
ASHGA5P014680	0.001152389	0.029181564	5.0614248	up	noncoding	ENST00000489284	RP5-1103I5.1	GENCODE

ASHGA5P039130	0.002368571	0.040102273	12.3344504	up	noncoding	ENST00000490357	RP11-10O22.1	GENCODE
ASHGA5P028211	0.003549114	0.048736439	2.7804379	up	noncoding	ENST00000501008	RP11-796E2.4	GENCODE
ASHGA5P044868	0.000335689	0.016627325	2.1656128	up	noncoding	ENST00000501104	RP11-159H10.3	GENCODE
ASHGA5P033943	0.000985807	0.026877506	2.3386886	up	noncoding	ENST00000501448	AC005329.7	GENCODE
ASHGA5P040338	0.002814751	0.043706406	2.102794	up	noncoding	ENST00000502209	RP11-308B16.2	GENCODE
ASHGA5P019809	0.001317957	0.030502257	2.4979197	up	noncoding	ENST00000503483	CTC-281B15.1	GENCODE
ASHGA5P019874	0.001871431	0.03536233	2.4953191	up	noncoding	ENST00000504474	RP11-65F13.2	GENCODE
ASHGA5P019876	0.005309266	0.059446578	2.1556458	up	noncoding	ENST00000504512	RP11-267A15.1	GENCODE
ASHGA5P052176	4.14384E-05	0.007762794	178.1430677	up	noncoding	ENST00000504989	RP11-325I22.2	GENCODE
ASHGA5P019955	0.000660409	0.022560077	5.1016427	up	noncoding	ENST00000505841	CYP4Z2P	GENCODE
ASHGA5P019980	0.006445103	0.066135475	2.6002332	up	noncoding	ENST00000506247	RP11-159F24.1	GENCODE
ASHGA5P041557	0.000120448	0.011004879	2.3741682	up	noncoding	ENST00000507361	RP11-267A15.1	GENCODE
ASHGA5P052184	0.000209729	0.01338424	6.2448417	up	noncoding	ENST00000507599	CTD-2247C11.3	GENCODE
ASHGA5P040115	0.000106047	0.010443757	2.7753005	up	noncoding	ENST00000507933	RP11-614F17.2	GENCODE
ASHGA5P052331	7.44652E-05	0.009754381	2.8847092	up	noncoding	ENST00000507963	RP11-65F13.2	GENCODE
ASHGA5P020105	0.000640653	0.022058204	3.9731852	up	noncoding	ENST00000508286	LEF1-AS1	GENCODE
ASHGA5P020132	0.000315904	0.016257852	2.234083	up	noncoding	ENST00000508572	RP11-25H12.1	GENCODE
ASHGA5P020168	6.89598E-05	0.009485505	3.0487743	up	noncoding	ENST00000508973	RP11-823P9.1	GENCODE
ASHGA5P020200	0.006710935	0.067504568	2.2844869	up	noncoding	ENST00000509460	CRYBB2P1	GENCODE
ASHGA5P040464	0.001664174	0.033596042	2.2071522	up	noncoding	ENST00000510261	CTD-2116N20.1	GENCODE
ASHGA5P020252	0.002343144	0.039787767	3.4379263	up	noncoding	ENST00000510407	RP11-39K24.10	GENCODE
ASHGA5P020319	0.004719249	0.055703759	2.3680582	up	noncoding	ENST00000511495	CTC-281B15.1	GENCODE
ASHGA5P020380	0.006252295	0.065070179	2.426595	up	noncoding	ENST00000512571	CTB-73N10.1	GENCODE
ASHGA5P020411	0.000412371	0.018188155	2.7821687	up	noncoding	ENST00000513055	RP11-65F13.2	GENCODE
ASHGA5P041271	0.00986086	0.082913078	2.3008307	up	noncoding	ENST00000513175	CTD-2007H13.3	GENCODE
ASHGA5P027749	0.000506536	0.020148093	2.1467433	up	noncoding	ENST00000513358	RP11-253E3.3	GENCODE
ASHGA5P043197	0.004348742	0.05362109	2.2383414	up	noncoding	ENST00000513631	DLX6-AS2	GENCODE
ASHGA5P039747	0.003605766	0.04915466	8.1548047	up	noncoding	ENST00000514297	RP11-756P10.2	GENCODE
ASHGA5P027321	0.006205279	0.064813174	3.9480648	up	noncoding	ENST00000514503	RP11-834C11.7	GENCODE
ASHGA5P045743	0.000164987	0.012497432	3.3907267	up	noncoding	ENST00000515258	FAM27E3	GENCODE
ASHGA5P039900	0.001457939	0.031508822	3.1209337	up	noncoding	ENST00000515422	RP11-395I6.2	GENCODE
ASHGA5P020597	3.02704E-06	0.002799679	7.0797996	up	noncoding	ENST00000517346	CTB-78F1.2	GENCODE
ASHGA5P045003	0.00980332	0.082588703	3.2662217	up	noncoding	ENST00000517482	RP11-383J24.1	GENCODE
ASHGA5P047435	0.001599564	0.032940286	2.0976156	up	noncoding	ENST00000517854	RP11-131N11.4	GENCODE
ASHGA5P020648	0.000369175	0.017237336	4.8078394	up	noncoding	ENST00000517953	RP11-705O24.1	GENCODE
ASHGA5P033204	0.000580481	0.02090606	2.8690707	up	noncoding	ENST00000518420	RP11-304F15.6	GENCODE
ASHGA5P044850	0.000719422	0.023314628	5.9504076	up	noncoding	ENST00000519215	RP11-705O24.2	GENCODE
ASHGA5P044202	0.00038004	0.017287375	11.6619416	up	noncoding	ENST00000519691	RP11-150O12.3	GENCODE
ASHGA5P044848	0.003941917	0.05148962	5.3401742	up	noncoding	ENST00000519967	RP11-705O24.1	GENCODE
ASHGA5P044849	0.001221055	0.030074685	4.6438509	up	noncoding	ENST00000519990	RP11-705O24.1	GENCODE
ASHGA5P044450	0.00095657	0.02649501	2.7197135	up	noncoding	ENST00000520268	KB-1562D12.1	GENCODE
ASHGA5P020866	0.009485178	0.080786426	2.2758748	up	noncoding	ENST00000521097	RP11-30J20.1	GENCODE
ASHGA5P044396	0.001653002	0.033525736	2.3530775	up	noncoding	ENST00000521148	RP11-100L22.2	GENCODE
ASHGA5P053431	0.002785818	0.043560422	5.0990279	up	noncoding	ENST00000521501	RP11-705O24.1	GENCODE
ASHGA5P044264	1.3602E-05	0.004560378	6.6436573	up	noncoding	ENST00000521558	RP11-1081M5.1	GENCODE
ASHGA5P041467	0.00362793	0.049367939	2.0062861	up	noncoding	ENST00000521756	RP11-394O4.3	GENCODE
ASHGA5P020922	0.000166482	0.012519564	2.9575124	up	noncoding	ENST00000521989	RP11-150O12.6	GENCODE
ASHGA5P045068	2.47833E-05	0.005996865	32.7326288	up	noncoding	ENST00000522374	AC145123.2	GENCODE
ASHGA5P020951	0.001135236	0.028915317	3.4168122	up	noncoding	ENST00000522471	RP11-150O12.3	GENCODE
ASHGA5P044721	0.000324136	0.016362629	3.13026	up	noncoding	ENST00000522547	RP11-14I17.2	GENCODE
ASHGA5P021033	0.002671604	0.042858823	3.5553889	up	noncoding	ENST00000523692	RP11-325I22.2	GENCODE
ASHGA5P043570	0.005200944	0.058685781	2.1435517	up	noncoding	ENST00000524304	HOXA-AS3	GENCODE
ASHGA5P020860	0.004968833	0.057367142	2.0014382	up	noncoding	ENST00000524346	RP11-30J20.1	GENCODE
ASHGA5P048447	0.003489083	0.048205231	2.0054441	up	noncoding	ENST00000526487	RP11-839D17.3	GENCODE
ASHGA5P026298	0.000362537	0.017237336	3.7706503	up	noncoding	ENST00000527270	RP11-646J21.4	GENCODE
ASHGA5P048374	0.001803049	0.034907088	4.0104625	up	noncoding	ENST00000528660	RP11-736K20.5	GENCODE
ASHGA5P021257	0.001841376	0.035117113	2.5231646	up	noncoding	ENST00000528800	RP11-159H10.3	GENCODE
ASHGA5P026437	0.000290849	0.015726086	2.8351389	up	noncoding	ENST00000529451	RP11-780O24.1	GENCODE
ASHGA5P026681	0.003061648	0.045759201	2.4808546	up	noncoding	ENST00000530030	RP11-718B12.3	GENCODE
ASHGA5P048044	0.000780995	0.024297315	4.6344028	up	noncoding	ENST00000532748	RP11-45A12.1	GENCODE
ASHGA5P055146	0.000444789	0.018808535	4.8559475	up	noncoding	ENST00000533504	RP11-839D17.3	GENCODE
ASHGA5P026732	4.33547E-05	0.007797264	4.3461293	up	noncoding	ENST00000534891	RP11-712B9.2	GENCODE
ASHGA5P054218	0.00267945	0.042905685	2.0322657	up	noncoding	ENST00000543097	TTTY15	GENCODE
ASHGA5P055101	0.002792848	0.043560422	2.7748844	up	noncoding	ENST00000543150	RP11-712B9.2	GENCODE
ASHGA5P022031	0.004069608	0.052325251	2.0555909	up	noncoding	ENST00000547577	CTD-2311B13.7	GENCODE
ASHGA5P022060	0.001316282	0.030502257	4.0516381	up	noncoding	ENST00000548217	RP11-597A11.1	GENCODE
ASHGA5P029406	0.00016249	0.012416233	3.4157504	up	noncoding	ENST00000548261	RP11-597A11.1	GENCODE
ASHGA5P022087	0.000566641	0.020743466	3.4662873	up	noncoding	ENST00000548793	RP11-536C10.8	GENCODE
ASHGA5P022142	0.000919725	0.025927903	5.2827142	up	noncoding	ENST00000550272	RP11-315E17.1	GENCODE
ASHGA5P022172	0.000546129	0.020574009	3.0770752	up	noncoding	ENST00000551067	CTD-2311B13.1	GENCODE
ASHGA5P029405	0.001704747	0.034003838	2.8401181	up	noncoding	ENST00000551144	RP11-244H18.3	GENCODE
ASHGA5P029052	0.008794838	0.077818014	2.0898785	up	noncoding	ENST00000552028	CTD-2384A14.2	GENCODE
ASHGA5P055349	0.001291986	0.030418569	3.3993908	up	noncoding	ENST00000552639	RP11-804F13.1	GENCODE
ASHGA5P029524	0.000691205	0.022894499	2.7245797	up	noncoding	ENST00000554328	RP11-111A21.1	GENCODE
ASHGA5P022359	0.000173516	0.012519564	2.2096594	up	noncoding	ENST00000555186	CHEK2P2	GENCODE
ASHGA5P022387	0.006326426	0.065438148	2.502463	up	noncoding	ENST00000555924	RP11-1085N6.2	GENCODE
ASHGA5P029301	0.002424963	0.040629181	5.1967674	up	noncoding	ENST00000555975	CTD-3035D6.2	GENCODE
ASHGA5P019710	0.005816082	0.062702833	2.6175735	up	noncoding	ENST00000556904	AC068831.10	GENCODE

ASHGA5P022460	0.001269153	0.030252916	3.36399	up	noncoding	ENST00000557216	RP11-536C10.8	GENCODE
ASHGA5P048706	0.001619401	0.03315301	4.0520988	up	noncoding	ENST00000557976	RP11-643A5.2	GENCODE
ASHGA5P048831	0.001476218	0.031712298	2.4539779	up	noncoding	ENST00000558086	CTD-2240J17.1	GENCODE
ASHGA5P030754	3.53717E-05	0.006924106	5.2583049	up	noncoding	ENST00000558221	CTD-3094K11.1	GENCODE
ASHGA5P030435	0.000729376	0.023377847	3.2767005	up	noncoding	ENST00000560866	RP11-702M1.1	GENCODE
ASHGA5P031443	1.53617E-05	0.004560378	5.5618375	up	noncoding	ENST00000561511	LA16c-381G6.1	GENCODE
ASHGA5P031332	0.003308419	0.047014091	3.0861255	up	noncoding	ENST00000561719	RP11-18F14.1	GENCODE
ASHGA5P045022	0.009310171	0.079863025	2.0206138	up	noncoding	ENST00000561978	PCAT1	GENCODE
ASHGA5P044321	0.000284788	0.015481514	2.3419149	up	noncoding	ENST00000562490	RP11-102F4.3	GENCODE
ASHGA5P022834	0.006539598	0.066623453	2.11266	up	noncoding	ENST00000563823	AC004158.2	GENCODE
ASHGA5P032264	0.000793738	0.02448946	3.9707495	up	noncoding	ENST00000564549	RP11-209D14.2	GENCODE
ASHGA5P039466	0.003684761	0.04983005	2.4731943	up	noncoding	ENST00000564925	RP11-109G23.3	GENCODE
ASHGA5P035703	0.007282813	0.070372253	6.3033083	up	noncoding	ENST00000565044	RP11-734K21.5	GENCODE
ASHGA5P022903	0.000374555	0.017265254	5.9960002	up	noncoding	ENST00000565133	RP11-354I13.2	GENCODE
ASHGA5P039088	0.004869089	0.056723908	2.6721438	up	noncoding	ENST00000565554	RP11-145F16.2	GENCODE
ASHGA5P044445	2.89102E-05	0.00636351	4.3872381	up	noncoding	ENST00000565617	KB-1460A1.5	GENCODE
ASHGA5P048553	0.000256247	0.014691304	2.3182492	up	noncoding	ENST00000566448	CHEK2P2	GENCODE
ASHGA5P044263	0.000263492	0.015001833	17.1523431	up	noncoding	ENST00000566892	RP11-1081M5.2	GENCODE
ASHGA5P031450	0.006624412	0.066982302	2.0049006	up	noncoding	ENST00000566922	LA16c-385E7.1	GENCODE
ASHGA5P044062	0.000471392	0.019395401	2.131339	up	noncoding	ENST00000567210	AF067845.1	GENCODE
ASHGA5P031003	0.000228162	0.01387845	3.9702316	up	noncoding	ENST00000567369	CTA-363E6.2	GENCODE
ASHGA5P023100	0.000581633	0.02090606	2.0190034	up	noncoding	ENST00000569859	RP11-67H24.2	GENCODE
ASHGA5P023144	0.00429548	0.053489199	2.1125739	up	noncoding	ENST00000571138	AC012146.7	GENCODE
ASHGA5P032758	0.001202161	0.029920082	2.4852687	up	noncoding	ENST00000572876	RP11-74E22.3	GENCODE
ASHGA5P032733	0.006259117	0.065102288	2.2954574	up	noncoding	ENST00000574471	RP11-388C12.1	GENCODE
ASHGA5P031012	0.000512788	0.020221999	2.9768623	up	noncoding	ENST00000574654	CTD-2194A8.2	GENCODE
ASHGA5P032084	0.006413678	0.066000653	2.521984	up	noncoding	ENST00000574885	CTD-3060P21.1	GENCODE
ASHGA5P044460	0.001205855	0.029944323	3.7286495	up	noncoding	ENST00000577199	RP11-1C8.6	GENCODE
ASHGA5P033071	0.000537378	0.020574009	2.1890093	up	noncoding	ENST00000577328	CTD-2206N4.4	GENCODE
ASHGA5P023335	0.003002788	0.045176656	2.6917838	up	noncoding	ENST00000578664	RP11-838N2.4	GENCODE
ASHGA5P033891	3.29182E-05	0.006747078	6.4161939	up	noncoding	ENST00000578967	RP11-676J15.1	GENCODE
ASHGA5P023346	0.007021823	0.069115369	2.6446274	up	noncoding	ENST00000579007	RP11-838N2.4	GENCODE
ASHGA5P033462	0.000174314	0.012519564	3.0199898	up	noncoding	ENST00000580242	RP11-720L2.3	GENCODE
ASHGA5P023418	7.18163E-05	0.009573706	3.8189049	up	noncoding	ENST00000580564	RP11-676J15.1	GENCODE
ASHGA5P033014	0.000111295	0.010712588	2.1902897	up	noncoding	ENST00000581019	CTD-2349P21.5	GENCODE
ASHGA5P049941	0.000550731	0.020610304	2.5077783	up	noncoding	ENST00000581029	RP11-838N2.4	GENCODE
ASHGA5P023461	0.001438107	0.031372189	2.6618925	up	noncoding	ENST00000581442	RP11-838N2.4	GENCODE
ASHGA5P033700	0.009044785	0.079026205	2.3890047	up	noncoding	ENST00000581488	RP11-737O24.2	GENCODE
ASHGA5P049984	0.004226701	0.052993068	2.8434296	up	noncoding	ENST00000581856	RP11-534N16.1	GENCODE
ASHGA5P033463	0.00074535	0.02369615	5.1203362	up	noncoding	ENST00000582120	RP11-720L2.2	GENCODE
ASHGA5P033435	0.003854033	0.050867394	2.7960589	up	noncoding	ENST00000582558	RP13-650J16.1	GENCODE
ASHGA5P032316	0.000137846	0.011553371	2.148559	up	noncoding	ENST00000582881	RP11-68I3.11	GENCODE
ASHGA5P033310	0.000796651	0.024521655	2.3623185	up	noncoding	ENST00000582940	RP11-160O5.1	GENCODE
ASHGA5P033010	0.001161081	0.029274031	3.123125	up	noncoding	ENST00000583030	LRRC37BP1	GENCODE
ASHGA5P023556	0.001820624	0.035002818	2.0280395	up	noncoding	ENST00000583982	RP11-267C16.1	GENCODE
ASHGA5P023557	0.003272753	0.046982583	2.533283	up	noncoding	ENST00000583985	RP11-92G19.2	GENCODE
ASHGA5P033466	0.000323777	0.016362629	2.9395673	up	noncoding	ENST00000584679	RP11-806L2.5	GENCODE
ASHGA5P033034	0.005020089	0.057577345	2.0965712	up	noncoding	ENST00000584721	RP11-227G15.8	GENCODE
ASHGA5P023585	3.62469E-05	0.006961667	2.5543457	up	noncoding	ENST00000584916	RP11-277J6.3	GENCODE
